# Evolutionary and epidemic dynamics of COVID-19 in Germany exemplified by three Bayesian phylodynamic case studies

**DOI:** 10.1177/11779322251321065

**Published:** 2025-03-12

**Authors:** Sanni Översti, Ariane Weber, Viktor Baran, Bärbel Kieninger, Alexander Dilthey, Torsten Houwaart, Andreas Walker, Wulf Schneider-Brachert, Denise Kühnert

**Affiliations:** 1 Transmission, Infection, Diversification & Evolution Group (tide), Max Planck Institute of Geoanthropology, Jena, Germany; 2 Department of Archaeogenetics, Max Planck Institute for Evolutionary Anthropology, Leipzig, Germany; 3 Department of Infection Prevention and Infectious Diseases, University Hospital Regensburg, Regensburg, Germany; 4 Institute of Medical Microbiology and Hospital Hygiene, Medical Faculty, Heinrich Heine University Düsseldorf, Düsseldorf, Germany; 5 Center for Digital Medicine, Heinrich Heine University Düsseldorf, Düsseldorf, Germany; 6 Institute of Virology, University Hospital Düsseldorf, Düsseldorf, Germany; 7 Phylogenomics Unit, Centre for Artificial Intelligence in Public Health Research, Robert Koch Institute, Wildau, Germany

**Keywords:** Bayesian phylodynamics, birth-death, SARS-CoV-2, reproductive number, viral genomics, BEAST2, Germany

## Abstract

The importance of genomic surveillance strategies for pathogens has been particularly evident during the coronavirus disease 2019 (COVID-19) pandemic, as genomic data from the causative agent, severe acute respiratory syndrome coronavirus type 2 (SARS-CoV-2), have guided public health decisions worldwide. Bayesian phylodynamic inference, integrating epidemiology and evolutionary biology, has become an essential tool in genomic epidemiological surveillance. It enables the estimation of epidemiological parameters, such as the reproductive number, from pathogen sequence data alone. Despite the phylodynamic approach being widely adopted, the abundance of phylodynamic models often makes it challenging to select the appropriate model for specific research questions. This article illustrates the application of phylodynamic birth-death-sampling models in public health using genomic data, with a focus on SARS-CoV-2. Targeting researchers less familiar with phylodynamics, it introduces a comprehensive workflow, including the conceptualisation of a research study and detailed steps for data preprocessing and postprocessing. In addition, we demonstrate the versatility of birth-death-sampling models through three case studies from Germany, utilising the BEAST2 software and its model implementations. Each case study addresses a distinct research question relevant not only to SARS-CoV-2 but also to other pathogens: Case study 1 finds traces of a superspreading event at the start of an early outbreak, exemplifying how simple models for genomic data can provide information that would otherwise only be accessible through extensive contact tracing. Case study 2 compares transmission dynamics in a nosocomial outbreak to community transmission, highlighting distinct dynamics through integrative analysis. Case study 3 investigates whether local transmission patterns align with national trends, demonstrating how phylodynamic models can disentangle complex population substructure with little additional information. For each case study, we emphasise critical points where model assumptions and data properties may misalign and outline appropriate validation assessments. Overall, we aim to provide researchers with examples on using birth-death-sampling models in genomic epidemiology, balancing theoretical and practical aspects.

## Introduction

Next-generation sequencing and its applications to genomic surveillance strategies for pathogens have provided an indispensable avenue for studying and controlling infectious disease outbreaks in the context of public health.^1-3^ The significance of genomic surveillance has become particularly evident during the severe acute respiratory syndrome coronavirus type 2 (SARS-CoV-2) pandemic, where viral genomic information has informed public health decisions on national and international levels.^4-6^ Monitoring pathogen infections through genome-based methods not only allows for molecular characterisation and observation of evolutionary changes but also harbours the potential to retro- and prospectively evaluate spatiotemporal transmission dynamics.^7^ It can thus provide valuable insights for informing mitigation strategies.

In recent years, genomic epidemiological surveillance has increasingly employed phylodynamics – a concept pioneered by Grenfell et al.^8^ It encompasses an integrated analysis of immunodynamics, epidemiology and evolutionary biology and has recently particularly focussed on epidemiological aspects. Phylodynamic methodologies predominantly operate under the assumption that evolutionary and epidemiological dynamics occur on similar timescales, such that the branching events within the viral phylogenetic tree correspond temporally to transmission events. Consequently, monitoring pathogen genetic data across time enables inferences about past epidemiological trends, and numerous studies have successfully applied phylodynamic methods to a wide range of pathogens, including viruses and bacteria (for a review, see Baele et al,^9^ Rife et al^10^ and references therein).

Bayesian statistics are widely adopted in fields of data-based parameter estimation due to their straightforward quantification of uncertainty by generating probability distributions that represent our prior belief updated in light of the observed data.^11^ In Bayesian inference, these distributions, also called posterior probabilities, are calculated from the product of the probability of the data under the assumed model given the parameters (‘likelihood’) and the probability of the parameters (‘prior’). To obtain a normalised distribution, this product is divided by the model evidence, that is the probability of the data under the assumed model (‘marginal likelihood’). As the posterior probability can only be calculated analytically for very simple models, in more complex inferences, it is usually approximated by numerical methods. The most common of these methods is the Markov Chain Monte Carlo (MCMC) algorithm, which draws a representative set of samples from the posterior distribution. In Bayesian phylodynamic inference, we usually aim to estimate posterior distributions for the tree, which reconstructs the ancestral relationship between the sequences, jointly with evolutionary and epidemiological model parameters. By treating the tree as a parameter of interest, the likelihood is calculated from the genomic data through an evolutionary model and a prior probability distribution for the tree is defined by a population dynamic process. In epidemiological applications, these population dynamic models are often chosen to directly quantify parameters of interest, like the growth rate or reproductive number (R) .^12^ The reproductive number corresponds to the average number of infections directly caused by 1 infected individual. We differentiate between the basic and effective reproductive number $\left(R_{e}\right)$ , on the basis of the immunity of the underlying population: the former assumes a naive population and is, therefore, used at the start of a new epidemic. The effective reproductive number relaxes this assumption and is thus quantified in the presence of, for example, interventions. Traditionally, the reproductive number is calculated from time series data of infection counts combined with assumptions on, for example, the serial interval.^13,14^ As the phylodynamic approach offers means to combine sophisticated population dynamic models with evolutionary reconstructions, it has led to the growing adoption of the Bayesian framework within the fields of phylogenetics and phylodynamics. Consequently, various software tools employing Bayesian inference have been implemented.^15^ In this article, we, however, only focus on the software BEAST2.^16^

Typically, the prior probability distribution of the tree is derived from birth-death processes^17,18^ or coalescent theory.^19^ In this article, we consider transmission dynamics in the context of a birth-death process with sampling through time. A more detailed description of coalescent-based models can for example be found in Featherstone et al.^20^ Within the birth-death-sampling framework, a bifurcating tree is generated forwards in time by allowing lineages to undergo birth/transmission, death/recovery and sampling events. Death/recovery events refer to the removal of an individual from the pool of infected individuals, either through death or recovery. In addition, an individual is assumed to be removed from said pool when being sampled. The corresponding rate parameters – that is the transmission rate λ, recovery rate μ and sampling rate ψ – allow the direct quantification of the following epidemiological quantities: the effective reproductive number $R_{e}=\frac{\lambda }{\mu +\psi },$ the rate to become noninfectious δ=μ+ψ and the sampling proportion $s=\frac{\psi }{\mu +\psi }.$ Within the given model specification, an individual is assumed to become noninfectious at the time of sample collection. Model extensions exist that relax this assumption and allow the individual to be removed from the population after sampling with a given probability.^21^ To use this process in Bayesian inference, a probability distribution for the sampled phylogenetic tree given the process has to be constructed. In short, this is done by defining differential equations that describe the probability of a change in the number of individuals in the process with respect to time. Practically, the sampled tree is then traversed from tip to root and the full probability is calculated as the product of the probability of the individual branches using the solution of the differential equations.

Due to the interdependence of the rate parameters, not all three epidemiological quantities can be estimated at the same time. From a mathematical perspective, this problem is usually discussed in the context of model identifiability. For birth-death models, it has recently been studied in more detail to provide a sound theoretical foundation for the inference problem and better guide practical inferences.^22-27^ It is important to note here that theoretical identifiability has not been shown for all birth-death models, including the one introduced above. Examples for identifiable models include birth-death without sampling through time or sampling through time without removal of the sampled individual from the group of infected individuals. However, even if the theoretical identifiability of a model is proven, the practical inference of it from a limited amount of genetic data remains challenging. It is, therefore, strongly advisable to scrutinise the model setting and inference results, for example through sensitivity analyses and simulation studies as discussed below. In phylodynamic inference, birth-death-sampling models are appealing as they allow for stochastic changes in the population size and explicitly model the sampling process. Particularly at the start of an outbreak, during its phase of exponential growth, models based on birth-death-sampling processes have been shown to outperform alternatives.^28^ In the full analysis, the inferred branching times of the phylogenetic tree are informed by both the genetic differences between sequences, through the evolutionary model, and their sampling dates, through the birth-death-sampling model. We will, therefore, refer to the posterior trees that are informed by the phylogenetic and population dynamic model as ‘phylodynamic’ trees. Moreover, certain model extensions facilitate the integration of intricate population dynamics by enabling changes in the epidemiological parameters through time over one or multiple subpopulations. More details on these models and their implementations in BEAST2 packages will be given in later sections.

In this article, we illustrate the use of phylodynamic birth-death-sampling models in extracting information relevant to public health from genomic data. For this, we focus on genomic data sets of SARS-CoV-2 that present a well-defined setup for phylodynamic analysis. We provide a detailed description of key considerations involved in using birth-death-sampling models with SARS-CoV-2 data, particularly intended for researchers with little experience with these methods. We start by describing the general conceptualisation of a research study utilising phylodynamics, followed by a section introducing technical details of a data analysis encompassing crucial steps of data preprocessing and postprocessing of the results. As an illustration, we present three case studies from Germany exemplifying how surveillance of genomic variation through time can be used to make inferences about the transmission dynamics of SARS-CoV-2. To demonstrate the versatility of birth-death-sampling models implemented in BEAST2 software packages, each case study addresses a distinct research question: Case study 1 evaluates whether viral genomic data alone can point towards a superspreading event as the epicentre of an outbreak. Case study 2 infers relative transmission dynamics to compare a nosocomial outbreak cluster and the surrounding community in the Düsseldorf area. Case study 3 quantifies regional transmission dynamics in south-eastern Germany in a complex substructured population. We furthermore interpret the applicability of the methods employed and the results for each case study within the context of public health. The article is structured in a way that the ‘General workflow – Conceptualisation’ section introduces the main conceptual parts of a Bayesian phylodynamic analysis, which, in principle, can be applied to the analysis of any pathogen. The subsequent section, ‘General workflow – Data analysis’, offers a summary of the technical parts and their rationale, with a focus on SARS-CoV-2. Case studies 1 to 3 delve into the epidemiological and methodological details of the analyses conducted in this article. They thus also target readers interested in the technical aspects of phylodynamic analysis for SARS-CoV-2.

## Methods

### General workflow – Conceptualisation

**Figure 1.**
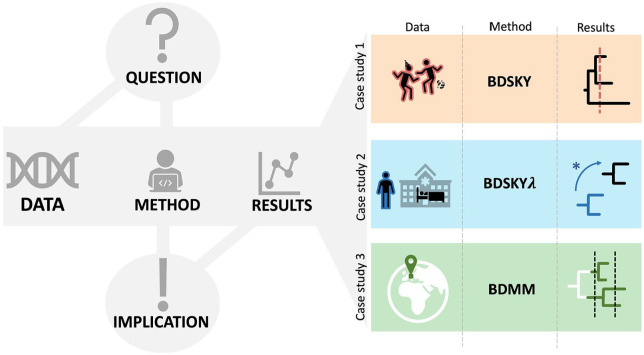
Bayesian phylodynamic studies: Main components and case study details. Left panel: The defining parts of the analysis workflow based on the research question, which addresses the specific epidemiological setting. Ideally, the data is collected under a well-defined strategy and a method is chosen that can resolve the main epidemiological drivers and yield the results of interest. By interpreting the results in consideration of the method’s assumptions, implications for public health strategies can be derived. Right panel: In each case study, we consider a distinct epidemiological context and present the type of data, method and result that can objectify it. We focus on birth-death-sampling models implemented in the Bayesian inference software BEAST2.

As in any scientific research, the process of generating new insights into the emergence and spread of infectious diseases within a Bayesian phylodynamic framework begins with formulating adequate **research questions** (Figure 1). These questions should be substantiated by the chosen **data and methodology**, ensuring that the data provide reliable information and the method directly contributes to understanding the intended outcomes of the study. In addition, it is necessary to obtain a baseline set of metadata associated with clinical samples intended for genome-based surveillance, which should include, at minimum, the sampling date and location.^29^ During the conceptualisation phase, special attention should be given to the potential impact of the sampling strategy on the results. Accurate modelling of the sampling process is crucial for inferences drawn from birth-death-sampling processes,^30^ as the sampling strategy has been shown to significantly impact the subsequent inference within the phylodynamic framework, including for SARS-CoV-2.^31^

In Bayesian phylodynamic analysis, selecting an appropriate method includes choosing an evolutionary and epidemiological model and defining prior distributions for parameters included in the model. Here, we will briefly discuss the selection of the epidemiological model, whereas the other aspects are covered in the section ‘General workflow – Data analysis’. The epidemiological model defines a probability distribution for the tree topology and branch lengths, the so-called ‘tree prior’. The models used in this study fundamentally rely on the same tree-generating process, a birth-death model with serial sampling, that can capture distinct aspects of transmission dynamics through model extensions. For example, the birth-death skyline model,^32^ as implemented in the BEAST2 package BDSKY, allows the rate parameters – transmission, recovery and sampling – to vary in a piecewise constant manner over time. This facilitates the reconstruction of changes in epidemiological parameters, particularly after specific points in time, and provides a framework for estimating the impact of nonpharmaceutical interventions on disease transmission.^33,34^ The BDSKYλ model extends the BDSKY model to allow the direct estimation of transmission differences between outbreak clusters relative to one baseline transmission rate.^35^ This method can be applied, for instance, to quantify the transmission advantage of one viral lineage over another. Importantly, both models assume each cluster to contain a panmictic population in which all infected individuals follow the same transmission dynamics. Especially in the case of a pandemic, this assumption is often violated because of population substructure, induced, for example, through transmission heterogeneity caused by superspreading or distinct subpopulations due to geographic barriers. Scientific questions seeking to quantify transmission patterns within and among such subpopulations can utilise a multitype birth-death model, available in the birth-death-migration model (BDMM) package.^36^ In this model, the parameters may vary among discretized subpopulations (‘types’) and, similar to BDSKY, can change over time in a piecewise constant fashion. Moreover, subpopulations can be connected through type-change events reflecting, for instance, a migration of an infected individual.

In a carefully predetermined context, the **results** of a phylodynamic analysis will directly align to the central research question(s). The primary outcome of the inference will be posterior samples of the parameters of interest – such as R, δ or s – as well as the phylodynamic trees. These represent a collection of plausible parameter values or time-scaled phylogenies each sampled proportional to their posterior probability. Within the Bayesian framework, uncertainties in inferred parameter estimates are characterised by probability distributions. Typically, one reports the mean or median estimate alongside a 95% highest posterior density interval (95% HPDI), which represents the narrowest parameter range that is covered by 95% of the posterior probability. The efficacy of the MCMC algorithm can be evaluated through its convergence rate and mixing efficiency. Convergence refers to the MCMC chain reaching a stationary phase, thereby approximating the true posterior distribution; typically, parameter estimates acquired before convergence are discarded as burn-in. Conversely, ‘mixing’ characterises how effectively the MCMC sampler explores the parameter space. In phylogenetic contexts, the convergence and mixing of the MCMC chain are often assessed through trace plots and effective sample size (ESS) estimates for each parameter in the model. Trace plots show the sampled parameter value against the respective MCMC state and, therefore, offer a visual tool to evaluate the length of the burn-in phase and the efficiency of mixing, whereas the ESS quantifies the number of independent samples required to achieve a similar level of precision under random sampling conditions. Generally, ESS values above 200 are considered indicative of convergence and representative posterior sampling in Bayesian phylogenetics (for discussion, see for example Nascimento et al).^15^ In addition, a common strategy to ensure MCMC sampler performance involves running the algorithm multiple times to verify that independent chains, that is chains that walk through the parameter space in a different way, converge to the same stationary distribution. Further technical insights into phylogenetic inference with MCMC and troubleshooting guidelines can be found, for example, in Barido-Sottani et al.^37^

Before using the sequencing data in the described Bayesian inference framework, it is advisable to run prior predictive simulations (also called ‘sample from prior’). In these analyses, samples are drawn from the full prior distribution only. The full prior is the product of the tree prior, calculated from the specified birth-death-sampling process, and all other prior distributions set on estimated parameters.^11^ As the sampling process is explicitly modelled in birth-death-sampling tree priors, these analyses are still informed by the observed data through the sampling times of the sequences. It is important to sample from the full prior for mainly two reasons: First, to assess that the MCMC chain is mixing well and can sample correctly from the prior distribution. This is verified through convergence of the ‘sample from prior’. Second, to validate that the joint prior distribution is sensible. Through parameter interactions, the actual prior distribution on a parameter can differ from the prior distribution that is defined for it during the analysis setup. Prior predictive simulations can, therefore, be utilised to inspect that these interactions do not lead to inappropriate full prior distributions.

**Implications** drawn from the outcomes of the phylodynamic framework can inform public health strategies for pandemic responses, as evidenced by various successful implementations during the SARS-CoV-2 pandemic.^38,39^ Whereas the main objective of employing phylodynamic tools on pathogen data is to enhance comprehension, management and forecasting of disease spread, it is crucial to recognise that the selected model(s) will also shape the resulting implications. Consequently, the interpretation of the results should be approached with the awareness that fitting a model to the data set involves simplifications, as the model can only describe the aforementioned processes within its specific limitations. Although ongoing research is dedicated to comparing model performance under certain epidemiological and evolutionary conditions,^28,30,40^ not all limitations of individual models are fully comprehended.

Therefore, it is crucial to assess the sensitivity of the results and the validity of the model choice: The robustness of the results against small changes in the analysis setup should be evaluated through sensitivity analyses. These analyses can involve, for example, using different model setups or prior distributions. Alternatively, re-estimating parameters of interest under the same model specification, but using a slightly different data set obtained through random subsampling or holding back specific samples, can provide insights into the sensitivity of the results towards certain data points. In addition, as detailed above, it is helpful to compare the resulting posterior distributions with the joint prior distribution. This comparison can reveal the extent to which the data influence the results through the phylogenetic likelihood, as well as the impact of the model assumptions through the population dynamic model. To evaluate the practical identifiability of a model for a specific research question, simulation studies are recommended. Through simulations, it is possible to assess the performance of the method under various model settings and/or data properties to improve the interpretability of the results. In principle, the efficiency of the Bayesian phylodynamic framework in informing public health policy and practice hinges on the careful design of research studies and the selection of validated models. By conducting rigorous evaluations of the results and model choice, researchers can enhance the credibility of their findings and eventually contribute to the control of infectious disease outbreaks in the context of public health.

### General workflow – Data analysis

**Figure 2.**
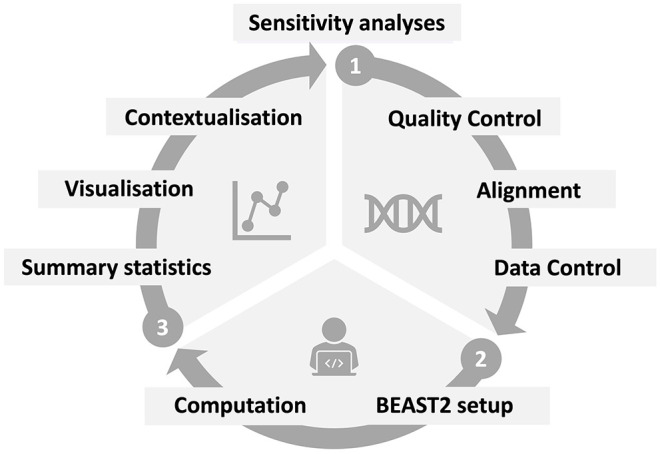
Overview of the steps included in a phylodynamic data analysis: Data-specific preprocessing steps include quality control (eg exclusion of low coverage genomes), multiple sequence alignment (MSA) and data control (eg masking of phylogenetically uninformative or misleading positions). In the main phylodynamic setup, the method specifications are defined (here in BEAST2) and the computation performed. To present the results of these computations, summary statistics of the Bayesian posterior sample have to be calculated, visualised and contextualised to obtain an interpretation as answer of the initial scientific question. Additional analyses to test the effect of changes in strong assumptions made during the BEAST2 setup, so-called sensitivity analyses, are important to demonstrate the robustness of the obtained results.

In Figure 2, we show a general workflow representation of the steps needed for a full phylodynamic analysis of SARS-CoV-2 data using the software package BEAST2. Starting from a genomic data set, we subdivide the steps into three parts: (1) preprocessing, (2) phylodynamic setup and (3) postprocessing. Preprocessing consists of steps that ensure suitable data quality and knowledge of its structure. The phylodynamic setup entails the definition of the full Bayesian model. Postprocessing steps are necessary to summarise the Bayesian posterior samples and visualise the resulting information. In the following, we will discuss all generalisable steps, that is parts (1) and (3) in full and part (2) with a focus on the evolutionary model. As such, we applied these in the same manner to all three case studies. As the epidemiological model in part (2) is informed by the specific scientific question, we discuss these steps for each case study separately.

#### Preprocessing

For case studies 1 and 2, we have obtained genomic SARS-CoV-2 sequence data sets of interest together with metadata from Walker et al,^41,42^ respectively. For case study 3, data were collected and sequenced at the University Medical Center Regensburg and at the MVZ Labor Passau (Supplemental Text S5). Similar data sets can be downloaded from public databases for sharing genomic surveillance data, like Global initiative on sharing all influenza data (GISAID)^43-45^ and National Center for Biotechnology Information (NCBI).^46^ The metadata associated with the data sets consisted of geographic location and sample collection date. During the **quality control** step, we excluded genome sequences with less than 29 001 nucleotides or more than 5.0% ambiguous data (eg uninformative characters ‘N’ or sequence alignment gaps) from the analysis because these characteristics can lead to inaccurate estimates of tree topology and branch lengths within the Bayesian framework.^47^ Moreover, we excluded genomes from further analyses if the collection date was incomplete, that is did not contain information about year, month and day, or did not lie within the time interval considered for analysis.

To compare the sequences site-by-site, we constructed multiple sequence alignments (MSA) using MAFFT v7.453.^48^ We used the first reported SARS-CoV-2 genome MN908947.3 as a reference and set the ‘keeplength’ and ‘addfragments’ options to decrease the computational demand. Alternative MSA tools are, for example, described by Pervez et al.^49^ As phylogenetic analyses are often purely based on information transported by the MSA, its quality can strongly impact the results.^50^ SARS-CoV-2, however, has only recently spread to the human host and has in the considered data sets not shown drastic genomic changes (ie longer insertions and duplications). The construction of MSAs for it is, therefore, still relatively robust, with different methods yielding comparable results.^51^ As a quality control measure for the MSAs, we, therefore, opted for visual inspection with Jalview v2.11.4.0.^52^

SARS-CoV-2 sequencing data have been shown to contain nonrandom errors associated, for example, with certain laboratories or sequencing protocols.^53,54^ Therefore, we performed additional **data control** to ensure that our sequence data only include information that is informative for the phylogenetic reconstruction and masked problematic sites, that is by not considering sites affected by sequencing errors or changing the nucleotide to an uninformative ‘N’.^53^ For case studies 1 and 2, which include data sets of single, clonal outbreaks, we further assessed the mutational sequence profile to ensure the clonal nature of all included genomes. Determining the clonal origin of an outbreak solely from sequence data can pose challenges, largely depending on the reference data utilised and/or the accessibility of patient information. Nonetheless, ruling out a suspected clonal outbreak can be guided by lineage classifications, as differing classes can point towards different epidemiological histories. For the lineage classification, we used the Pango lineage^55^ nomenclature. For the clonal outbreaks, we also built maximum likelihood phylogenetic trees and checked for phylotemporal outliers in TempEst,^56^ as we assume genomic changes to occur through time with a constant rate (‘strict molecular clock model’). A phylotemporal outlier is characterised by distinctly more or less genetic changes than expected given its sampling date. One way to identify them is via the residuals of the linear regression (https://beast.community/tempest_tutorial, last visited May 16, 2024).

#### Phylodynamic setup

To define the phylodynamic model, key components include defining an epidemiological model to characterise population dynamics, selecting an evolutionary model to describe sequence evolution, specifying prior distributions for parameters in these models and, finally, deciding on the number of iterations and samples for the MCMC process. For each case study, the epidemiological model and related **BEAST2 setups** are defined individually, based on the scientific question and data set, and are detailed in the following sections. As explained above, not all parameters in the epidemiological model can be estimated simultaneously. To obtain reliable estimates, we opted to inform the inferences through realistic prior assumptions and test these through sensitivity analyses. In doing so, we follow recommendations by Louca et al^22^ to fix at least one parameter for the entire period covered by the tree and another parameter in at least one time interval to obtain theoretical identifiability. For practical identifiability, we explore limitations of the model through simulations, as suggested by Legried and Terhorst.^23^ To estimate the effective reproductive number, we assumed the rate to become noninfectious to be known by fixing it to a literature estimate, that is 36.5 years^-1^, translating to an average infectious period of ten days.^57-62^ In addition, we constrained the sampling proportion through either bounded or narrow prior distributions. Below, we provide a brief overview of the substitution and clock model selection with a focus on SARS-CoV-2 data followed by the description of the MCMC settings. A more comprehensive introduction to these concepts can be found in the studies.^15,37,63,64^

The molecular evolutionary process that generated the sequence data can be described mathematically through a nucleotide substitution and evolutionary clock model. Selecting a substitution model that accurately fits the data is crucial, as wrong assumptions about the mutational process can lead to systematic errors in phylogenetic reconstruction.^65^ In general, two approaches can be employed for substitution model selection: first, using a priori knowledge of the data to choose an appropriate model and, second, employing likelihood-based model selection or averaging algorithms, such as jModelTest2,^66^ PartitionFinder^67^ or bModelTest.^68^ In our study, we reconstructed phylogenetic trees for individual phylogenetic lineages with highly identical sequences. Given the simplicity of these evolutionary relationships, we opted for the first approach and chose to utilise the Hasegawa-Kishino-Yano-85 (HKY85) model,^69^ which estimates base frequencies and accounts for the difference between transitions and transversions through a scaling factor. Moreover, among-site rate variation was modelled through a gamma distribution (Γ).^70^ The same approach, involving the selection of the HKY + Γ substitution model based on prior knowledge, has been commonly employed in previous SARS-CoV-2 studies.^34,35,59,71,72^

Sequence data from recent outbreaks often lack enough evolutionary changes over time for robust inference of evolutionary rates.^71^ To improve the accuracy of phylodynamic reconstructions for SARS-CoV-2, it is common to assume clock-like evolution and restrict the rate of evolution by previous knowledge.^34,35,59,72^ In our case studies, we focused either on a single clonal outbreak (case studies 1 and 2) or reconstructed phylodynamic trees for each Pango lineage separately (case study 3). Given the lack of strong evidence of nonclock-like evolution within lineage and the low genetic diversity, we followed the approach from studies^34,35,59,72^ and used a strict clock model to describe the accumulation of diversity over time, fixing the rate to a well-established value of 0.0008 substitutions/site/year.^73^ These assumptions, however, can greatly impact the temporal scale of the phylodynamic trees. To illustrate this impact, we provide additional analyses relaxing the assumptions for case study 1, which focuses on timing the first transmission event of the outbreak (see section ‘Case study 1 – Superspreading during carnival’). For SARS-CoV-2 data sets with higher levels of genetic diversity, evaluating the temporal signal and selecting the best-fit clock model are critical steps in molecular dating and phylodynamic inferences.^71^

For **Bayesian computation**, we adjusted the MCMC chain length according to data set size and sampled every 10 000th iteration. For each case study, we condition the birth-death-sampling process on the time of the root node. Moreover, as described above, for each presented data analysis, we opted to first sample from the full prior distribution using the same settings as for the Bayesian inference. For all data analyses, we ran at least two replicates with different seeds, that is initialisations of the random number generator, and used Tracer v1.72^74^ to assess convergence. If all replicates individually converged and their joint posterior distributions agreed with one another for all parameters, with ESSs of at least 200, we continued with the postprocessing steps.

#### Postprocessing

To calculate the **summary statistics** for the resulting posterior samples, we distinguish between single parameters and the tree structure: For the posterior sample of phylodynamic trees, we construct a maximum clade credibility (MCC) tree in TreeAnnotator v2.6.3, a tool available as a part of the BEAST2 installation. We set a posterior limit of 0.0 and burn-in percentage of 10.0 and selected the median to represent node heights. For single parameters, we use Rstudio^75^ to calculate the quantiles, particularly the median, and 95% HPDI. To **visualise** the results, we use custom R scripts, employing functions from the packages ape,^76^ ggtree,^77^ ggplot2^78^ and utile.visuals.^79^ To provide **context** for our results, we present them together with available metadata or epidemiological information.

Since these analyses require a set of assumptions in each specific model setup, we also ran multiple **sensitivity analyses** for each case study. These included, for instance, utilising less stringent prior distributions on the sampling proportion or relaxing the assumption of clock-like evolution. Furthermore, to assess the performance of each method in identifying the specific parameters of interest in each case study, we used simulations. We discuss the sensitivity analyses as well as the simulation studies shortly below and in more detail in the Supplemental Materials.

## Results

### Case study 1 – Superspreading during carnival

#### Epidemiological context and data

One of the first larger outbreaks of coronavirus disease 2019 (COVID-19) that was detected in Germany happened in February 2020 in the district Heinsberg. After the diagnosis of the first case on the February 25, 2020, local COVID-19 case numbers quickly started to rise. Through epidemiological investigations, many of the early infections were traced back to a large-scale carnival event on February 15, 2020.^41^ After this initial superspreading event, infections with SARS-CoV-2 continued to be diagnosed in the area despite early nonpharmaceutical interventions.^80,81^ Phylogenetic analyses of viral genome data sampled until April 2020 showed that at the end of the sampling period the outbreak already consisted of two distinct genetic groups. The first of these includes all early sequences associated with the initial superspreading event, which are assigned to Pango lineage B.3. The second was first sampled in mid-March and includes sequences from multiple different Pango lineages.^82^ This data set, therefore, presents an example of the seeding dynamics of a disease in a naive population. It highlights the interplay between early high-transmission events and multiple independent introductions in continued onward transmission.

With this case study, we aim to investigate whether we can quantify these seeding dynamics from genomic data. The genomic data, in this case, consist of viral sequences sampled in the Heinsberg area over a period of two months after the first local COVID-19 detection. The sequences form two subgroups^82^: samples from the initial connected outbreak and epidemiologically unconnected samples from multiple Pango lineages. In the following, we will focus on the former subgroup, because the latter has a very small sample size and is likely affected by nonpanmictic transmission dynamics. As we are interested in estimating the timing of the first transmission event in the first subgroup, as well as the following transmission dynamics, we use the BDSKY model. Through the joint inference of the transmission dynamics with a time-scaled phylogenetic tree, it allows us to date the most recent common ancestor (MRCA) of all included samples. We can thus draw conclusions about the timing of the initialisation of the outbreak. To be able to detect the overdispersed transmission dynamics at the start of the outbreak, we model a change in the transmission rate directly after the superspreading event. It is important to note that this model does not directly describe superspreading dynamics, as it estimates population-average transmission dynamics. However, as the first interval is very short and mainly includes the carnival event, the population size of transmitting individuals included in this interval is relatively small. We, therefore, expect to detect a population average that is elevated to values higher than the basic reproductive number in the region, if the data is informative for a superspreading event. Other, more complex, phylodynamic models exist that explicitly account for heterogeneity in the transmission dynamics.^38,83-85^ Due to the relatively small sample size and the high phylogenetic uncertainty, we chose the simpler model in a trade-off between model correctness and robustness of estimates.

#### Phylodynamic setup

We started with an initial set of 125 sequences sampled between February 25, 2020, and April 20, 2020. After the data preprocessing, as described above, 47 sequences, assigned to Pango lineage B.3, remained. Due to the epidemiological context, we opted for a stricter quality control in this case study: as we expect only very few correctly informative sites in the data set, sequencing artefacts can potentially have a bigger impact. For the main analysis, we thus excluded sequences with more than 1.0% Ns and more than 0.05% unique amino acid mutations (as recommended in GISAID).^44^ The first collection date in this data set was February 25, 2020, and the last April 6, 2020. We used the panmictic birth-death-sampling model implemented in BDSKY. We allowed the reproductive number to change on February 16, 2020, the day after the potential superspreading event. For both intervals, we used the prior probability distribution LogNormal(0.0, 4.0) (Supplemental Table S1). We fixed the sampling proportion to zero for the time interval before the first sample collection, as no sampling efforts were implemented in the region before this date. We set a strong prior distribution, that is Beta(32.0, 968.0), for the following time interval. This defines a prior mean of 0.032, which corresponds to the ratio of included genome samples and number of diagnosed infections until April 6, 2020, in the district Heinsberg, that is $\frac{47}{1474}$ .^86^ The MCMC chains were run for $8\times 10^{7}$ steps each. To test the robustness of the results against changes in the assumptions about the epidemiological parameters, we ran 5 sensitivity analyses (see Supplemental Table S2). Sensitivity analysis 1 includes all genomes passing our general quality control steps and analysis 2 sets a less strict prior on the sampling proportion. For the former, we adjusted the prior on the sampling proportion accordingly to Beta(71.0, 929.0), with a mean of $\frac{121}{1698}≃7.1\%.$ Sensitivity analyses 3 to 5 relax the assumption of strict clock-like evolution with fixed rate by using an uncorrelated log-normal relaxed molecular clock (UCLD) model (sensitivity analysis 3) and estimating the strict clock rate with a narrow prior (sensitivity analysis 4-5). All prior distributions are reported in Supplemental Table S1. To further evaluate the practical identifiability of the epidemiological parameters from our model setup with simulations, we focused on two setting: as a positive control, we simulated data with a pronounced drop in the reproductive number shortly after the start of the process and as a negative control we used our model setup on data that were generated with a constant reproductive number. Details are given in Supplemental Text S1.

#### Results and interpretation

**Figure 3.**
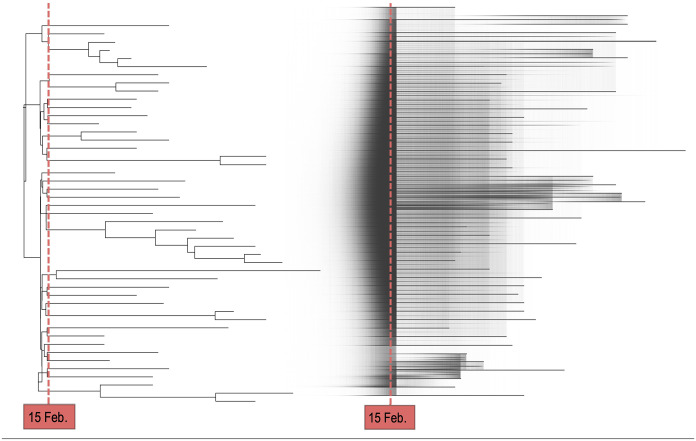
Case study 1 – Superspreading: MCC tree using median node heights of genomes belonging to Pango lineage B.3 (left panel). Posterior sample of consensus trees visualised with DensiTree^90^ (right panel). As in this illustration all posterior samples are plotted on top of each other, darker shaded areas highlight a high edge/node density or uncertainty. The reproductive number is allowed to change shortly after the carnival event on February 15, 2020 (the red dashed line).

In the estimated phylodynamic summary tree (Figure 3, left panel) we see, first, the timing of the branching events and, second, the topology, that is how the sequence samples cluster together and coalesce back in time. From the estimated branching times, we can conclude the following: With 95% of probability, the first transmission event of the outbreak happened within the 11 days preceding the carnival event, that is between February 4 and February 15, 2020. Most of the reconstructed transmission events occur temporally close to the carnival event. This strongly supports the outbreak to have started shortly before the carnival event and to have grown substantially around the time of the event. This becomes even more evident from the full posterior sample of phylodynamic trees (Figure 3, right panel), which shows distinctly higher edge density until directly after the carnival event. The sharp decrease in edge density coincides with the time when the reproductive number is allowed to change, as the reproductive number and the number of branching events in a time interval are correlated. The high edge density thus highlights how strongly the data supports most transmission events to have happened in the first interval. From the topology of the estimated tree, we can distinguish two clades that are potentially of epidemiological relevance: The first transmission, with high certainty, gave rise to two distinct sublineages, one consisting of 12.7% of included sequences, the other of 87.3%. Although most branching events of the bigger group are dated around the time of the carnival event, fewer branching events of the smaller group have 95% HPDI that include the event. This suggests that after the initial transmission event, one large subgroup got infected mainly during the carnival session, whereas another grew a few days later. The increased transmission during the carnival event is reflected in the estimates of the reproductive number: In the first interval, ending shortly after the carnival event, we estimate a median reproductive number of 15.48 (95% HPDI: [4.8, 41.5]). This estimate is higher than the population level basic reproductive number of SARS-CoV-2^87-89^ and strongly suggestive of a superspreading event. In the second interval, which spans roughly two months afterwards, the estimated reproductive number dropped to below one (median: 0.78, 95% HPDI: [0.61, 0.95]). The latter estimate highlights the declining dynamics of this outbreak after the carnival event. The validity of these findings is supported by the simulation results (Supplemental Table S3) and by all sensitivity analyses (Supplemental Table S2). The only remarkable difference we find is with sensitivity analysis 4, in which we estimate the substitution rate with a narrow prior. This results in a lower median estimate of the substitution rate which scales the tree height to higher values. As we fix the time point when the reproductive number is allowed to change to directly after the carnival event, the time covered by the first interval becomes longer. Thus, we estimate a lower reproductive number in the first interval, which now includes a much bigger population outside of the carnival event. However, if we instead let the reproductive number change ten days after the root node (sensitivity analysis 5), we again recover the elevated transmission rate. It is important to note here that we cannot quantify the superspreading behaviour directly but only the average transmission behaviour in the whole population over a time period and that the inferred time scales are generally highly sensitive to the value the substitution rate is fixed to. As mentioned above, however, prior information on the rate is necessary if the sequence data are not informative enough.

By definition, it is relatively unlikely that the MRCA of different Pango lineages falls in this region. Hence, the second subgroup most likely represents multiple independent introductions of SARS-CoV-2 into the area within the sampling period. In Supplemental Figure S1, we also show the summary tree for all samples assigned to Pango lineage B.1 in the second subgroup. Despite the high uncertainty in the estimated parameters for this lineage due to the small sample size and low genetic diversity, we see that it could have also been introduced during the carnival festivities in February.

Taken together, these results highlight the underlying smaller-scale dynamics that took place during the first weeks of COVID-19 spread in Heinsberg: Local case numbers first began to rise drastically due to a superspreading event. However, although the outbreak caused by this event started to decline, SARS-CoV-2 was introduced into the region on multiple other occasions independently. With this case study we thus demonstrate that from viral genome sequences and their collection dates we are able to reconstruct many important aspects of the epidemiological setting in this region. We provide strong indications for an initial superspreading event during the carnival session on February 15, 2020. We further show that the outbreak started shortly before this event and visualise further small-scale dynamics. Without expensive contact tracing efforts, conclusions like these are commonly not accessible from other surveillance data, like the number of hospitalisations or cumulative case counts.

### Case study 2 – Hospital outbreak

#### Epidemiological context and data

SARS-CoV-2 transmissions in healthcare settings have been found to play a crucial part in disease propagation,^91^ and despite the large number of reported nosocomial outbreaks (see Table 1 in the study by Abbas et al),^92^ genomic-based inference of transmission dynamics in healthcare settings has received little attention (for discussion, see Abbas et al^92^ and Shirreff et al^93^). Nevertheless, characterising viral spread within healthcare facilities holds immense potential for informing epidemic control policies. In this case study, we aim to determine whether transmission dynamics differ between a hospital outbreak and the surrounding community. We use nosocomial transmission chain data depicting an outbreak of lineage B.1.221 that emerged at Düsseldorf University Hospital in late 2020, affecting 29 patients and healthcare workers (‘Ward D’).^42^ A combined analysis of viral genomes from the hospital transmission chain and genomes from surveillance sampling revealed a highly clonal nature of the hospital outbreak.^42^ Although the joint analysis of viral genomes and contact tracing data by Walker et al^42^ identified potential infection clusters, our phylodynamic approach aims to further quantify relative transmission differences between the nosocomial outbreak and a nontargeted population surveillance sample. This is achieved using a baseline transmission rate and cluster-specific scaling factors, as implemented in the BDSKYλ model.

#### Phylodynamic setup

We utilised 23 SARS-CoV-2 sequences from the hospital outbreak and 50 sequences from the local background population. Surveillance sequences, also representing Pango lineage B.1.221, covered a timespan from August 17, 2020, to December 16, 2020, whereas the nosocomial outbreak was sampled between December 14 and December 23, 2020. For all samples, we performed the data preprocessing steps as outlined in Figure 2 and the corresponding text. For the hospital cluster, analysis with TempEst did not reveal any phylotemporal outliers.

Relative transmission rates between hospital and surveillance sample clusters were estimated with the BDSKYλ model. Within Bayesian inference, we inferred for both clusters a separate tree, sampling proportion and transmission rate ratio $(r_{\lambda }),$ while linking the baseline reproductive number ( $R_{base}$ ), the rate to become noninfectious and the substitution model, meaning that the latter parameters are shared between both clusters. The baseline reproductive number was allowed to change on November 2, 2020, when Germany enforced a partial lockdown (https://www.bundesregierung.de/breg-de/themen/coronavirus/regelungen-ab-2-november-1806818, last visited June 17, 2024). In both intervals, a LogNormal(0.0, 1.25) prior distribution was used for $R_{base}.$ The transmission rate ratio for the surveillance sample cluster ( $r_{\lambda ,surveillance}$ ) was fixed to 1.0, whereas for the hospital cluster $r_{\lambda ,hospital}$ was estimated by using a LogNormal(0.0, 1.0) prior distribution. The sampling proportions for both clusters were set to 0.0 before the first sample, that is before August 17, 2020, and before December 14, 2020, for surveillance and outbreak, respectively. Moreover, for the surveillance tree, we allowed a piecewise change in the sampling proportion on October 12, 2020, as the percentage of sequenced cases of all newly diagnosed infections declined substantially at that time^42^ (Supplemental Text S2 and Supplemental Figure S2). We chose sampling proportion priors based on estimates given by Walker et al,^42^ and thus, Uniform(0.0, 0.20) and Beta(3.1, 96.9) prior distributions were used for $s_{surveillance,t1}$ and $s_{surveillance,t2},$ respectively (Supplemental Text S2 and Supplemental Table S4). Accordingly, an upper limit for the prior distribution of $s_{hospital,t1}$ was defined based on the proportion of sequenced outbreak samples, and a Uniform(0.0, 0.80) distribution was used. We furthermore assessed the sensitivity of our results by re-estimating $R_{base}$ and $r_{\lambda ,hospital}$ under different prior distributions for $s_{surveillance,t1}$ and $s_{surveillance,t2}$ (Supplemental Text S3 and Supplemental Table S4). For the main and sensitivity analysis, we ran three parallel MCMC chains with $1\times 10^{7}$ steps each. To further validate the performance of the BDSKYλ model under transmission dynamics similar to those of case study 2, we conducted an additional simulation study. The simulation settings were designed to reflect the empirical data, and therefore, we simulated ‘surveillance’ and ‘hospital outbreak’ transmission trees under transmission parameters that corresponded to the results obtained from the Düsseldorf data. For further details on the simulation settings, see Supplemental Text S4.

#### Results and interpretation

**Figure 4.**
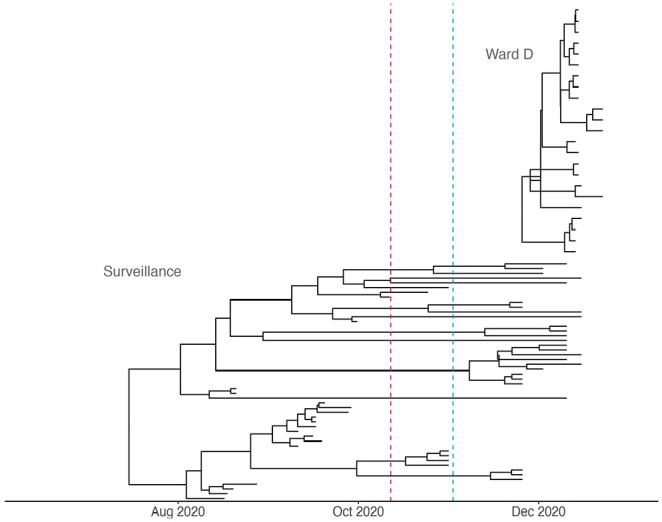
Case study 2 – Hospital outbreak: Phylodynamic trees inferred for the hospital outbreak cluster and surveillance sample cluster. The upper tree has been reconstructed based on viral genomes obtained from a nosocomial outbreak (‘Ward D’, N = 23), whereas the lower tree represents a nontargeted population surveillance sample (N = 50). For the surveillance data, the sampling proportion was allowed to change on October 12, 2020, indicated with a red dashed line, as the percentage of sequenced cases over all confirmed infections decreased (Supplemental Text S2 and Supplemental Figure S2). For the baseline reproductive number $(R_{base}),$ we allowed a piecewise change on November 2, 2020, indicated with a blue dashed line, as Germany enforced a partial lockdown. In the nosocomial outbreak tree, some branches near the root overlap, indicating that the inferred date for a descendant node is older than its direct ancestor (for details, see the main text and Supplemental Figure S3).

We estimated the baseline reproductive number within two time intervals: before and after the introduction of a partial lockdown in Germany. Despite the 95% HPDIs overlapping, the inferred baseline transmission rate estimate within the first interval was lower than the corresponding estimate for the second interval yielding median values of $R_{base,t1}=1.15$ (95% HPDI: [0.97, 1.35]) and $R_{base,t2}=1.48$ (95% HPDI: [1.21, 1.76]), respectively. The epidemic increase in the second interval, which coincided with more stringent intervention measures, could indicate that, whereas the partial lockdown presumably slowed SARS-CoV-2 spread in the Düsseldorf area, not all transmission chains were halted. A closer look at the SARS-CoV-2 strains circulating in the area reveals that at the beginning of November, lineage B.1.221 accounted for approximately 25% of new cases,^42^ and thus, this particular lineage cannot be considered representative of the overall transmission dynamics before or during the partial lockdown. It is, therefore, plausible that despite the lockdown reducing the overall number of new infections, as shown by Walker et al^42^ (and Figure 2A therein), lineage B.1.221 may have continued to spread faster than the remaining circulating lineages. Moreover, sustained transmission processes of B.1.221 with undetected cases could explain the extended branches seen in the surveillance tree (Figure 4). However, it is unlikely that the surveillance sequences are representative of samples from a single transmission cluster. Instead, the data likely consist of samples from several different transmission chains co-circulating in the area. Nonetheless, because in this study the data from the surveillance sample collection are considered to represent a baseline that averages the spread of B.1.122 in Düsseldorf, we chose not to perform additional analyses to identify independent transmission clusters. In the nosocomial outbreak tree, it is noteworthy that some descendant nodes near the root have older inferred dates than their direct ancestors, resulting in overlapping branches (Figure 4, upper tree). These negative branch lengths can occur in trees with branches that have very low posterior support. As the MCC tree is constructed using median node heights from posterior tree samples, poorly supported clades might not be present in the trees containing the ancestral clades. Consequently, the median heights for consecutive nodes are derived from different sets of trees, which can result in negative branch lengths. The clonal nature of the SARS-CoV-2 outbreak at Ward D means that the viral genomes share identical or nearly identical genetic profiles (Figure 3C in the work by Walker et al^42^), leading to lower posterior support for some branches within the phylodynamic tree (Supplemental Figure S3).

While the $R_{base}$ estimates suggested a general increase in the transmissibility through time, the cluster consisting exclusively of sequences from the hospital outbreak demonstrated even higher levels of transmission. The median estimate of the transmission rate ratio for the nosocomial cluster $\left(r_{\lambda ,hospital}\right)$ was 1.44 (95% HPDI: [0.85, 2.26]), indicating a ~1.5-fold transmission advantage of B.1.221 within the hospital as compared to the community. As the comparison between hospital and surveillance data is based on genetically highly similar genomes all representing the same Pango lineage, the estimated higher transmissibility within Düsseldorf University Hospital is presumably driven by extrinsic factors rather than, for instance, an increased viral fitness. Comorbidities among hospitalised patients that lead to increased susceptibility for SARS-CoV-2 infection may have amplified the number of healthcare-associated cases. As noted by Walker et al,^42^ inadequate use of preventive measures also likely played a significant role in the uncontrolled transmission at Ward D. Specifically, subsequent investigations revealed that inefficient medical mask wearing among both healthcare workers and patients, the latter due to various medical reasons, contributed to the size of the outbreak at Ward D. On a broader scale, the inferred transmission rate estimate for Ward D, which corresponds roughly to a reproduction number of 2.1, is consistent with previous estimates obtained across healthcare facilities.^93-95^

The inferred sampling proportions for the surveillance data were in accordance with the percentages of sequenced cases as reported by Walker et al.^42^ For the time periods covering calendar weeks 33 to 41 and 42 to 51, we inferred median estimates of $s_{surveillance,t1}=0.1$ (95% HPDI: [0.05, 0.18]) and $s_{surveillance,t2}=0.04$ (95% HPDI: [0.04, 0.19]), respectively. Both parameters were robustly inferred under different prior distributions (Supplemental Table S5). As asymptomatic carriers of SARS-CoV-2 infection may play an important role in disease transmission, also within healthcare settings,^96^ we chose a rather wide prior distribution of Uniform(0.0, 0.8) for $s_{t1,hospital}.$ The inferred posterior distribution of the nosocomial sampling proportion with a mean of 0.72 (95% HPDI: [0.50, 0.8]) suggests that most of the hospital-acquired infections were detected. Through sensitivity analyses, we further evaluated the robustness of the inferred transmission rate estimates and their sensitivity to the prior distribution used for the surveillance sampling proportion. As shown in Supplemental Table S5, estimates for $R_{base}$ and $r_{\lambda ,hospital}$ were robustly inferred within the sensitivity analysis where different $s_{surveillance}$ prior distributions were applied. Furthermore, a small-scale simulation study was conducted to confirm that the BDSKYλ model can robustly infer the underlying transmission dynamics under scenarios that mimic the characteristics of the Düsseldorf data (Supplemental Text S4). The results of the three simulated scenarios showed that the main parameters of interest were inferred with high accuracy and precision, providing additional support for the validity of the main results (Supplemental Text S4 and Supplemental Table S6).

In summary, given that both, nosocomial and surveillance data, encompass viral genomes of the same Pango lineage collected during a similar time frame, the elevated transmissibility observed within hospital settings likely correlates with environmental factors. Therefore, our results highlight the essential need to enforce stringent preventive measures within healthcare facilities to shield the most vulnerable individuals from infection. Rapid management of nosocomial outbreaks not only prevents transmissions among patients but also substantially influences epidemic dynamics in the surrounding community.^91^ Moreover, the characterization of a baseline transmission rate and its cluster-specific scaling factor in this study showcased the suitability of the BDSKYλ model for inferring relative disparities in transmission dynamics between multiple clusters. However, it is important to note that challenges for estimating the reproductive number can arise from the typically small sample sizes of nosocomial outbreaks and the stochastic nature of the transmission process.

### Case study 3 – Spatiotemporal pandemic excerpt

#### Epidemiological context and data

One important challenge in phylodynamic inference of regional transmission dynamics during a pandemic is population substructure due to multiple independent introductions. To address this problem, we focus on two research questions with this case study, one relating to methodology and one to epidemiological context: Can we successfully infer regional transmission dynamics from data sets with high levels of unknown substructure? Do the inferred local transmission dynamics align with the dynamics on the national level?

If a disease is introduced multiple times into the considered area, the genomic data sampled will likely not represent one clonal outbreak but multiple independent, simultaneously spreading transmission chains. The MRCA and many internal nodes of a phylogenetic tree reconstructed from all genome samples will, therefore, represent transmission events outside of the considered area. If the research goal is to quantify the regional transmission dynamics, these parts of the tree will bias the resulting estimates as they represent the epidemic dynamics outside of the region. Correctly treating these independent introductions becomes more difficult the higher the number of introductions and the more closely related the respective genome samples are. To account for this substructure, two main approaches exist: First, identifying the independent introductions to group the sequences into clusters belonging to the same outbreak and reconstructing a tree for each outbreak separately as in case study 1. Second, using a phylodynamic model that accounts for this substructure and reconstructing the global tree, as done in this case study. We consider a genome data set with high levels of unknown substructure. Through regional sequencing efforts, we obtained SARS-CoV-2 genome sequences of around 5% of diagnoses from multiple laboratories in south-eastern Germany sampled between January and October 2021. In Supplemental Figure S4, we show the number of sequences and diagnosed infections per Landkreis during the sampling period. This illustrates that the considered data set comprises samples that represent only a very small fraction of cases in a geographically not explicitly defined area. In addition, the sequences show high genetic similarity, even across time and space. Grouping into connected outbreaks through cluster detection methods is, therefore, difficult. We thus opted for the second option and used a population structure-aware phylodynamic model to infer the transmission dynamics.

#### Phylodynamic setup

We obtained 931 sequences from East Bavaria, sampled between January 13, 2021, and October 30, 2021. Details regarding the sample collection, sequencing and quality control are given in Supplemental Text S5. Each sequence included the collection date and first three numbers of the postal code as metadata. To reduce computational complexity of the Bayesian analysis, we inferred an independent tree for each Pango lineage with more than five sequences. To validate that the size of included Pango lineages does not change our results, we ran the same inference also on all Pango lineages with at least 20 sequences. We used pangolin^97^ to identify the lineages and ran the preprocessing steps as described above for each of the lineages. This resulted in 32 Pango lineages and 698 sequences in total. In a joint Bayesian analysis, we inferred independent phylodynamic trees for each Pango lineage with a shared effective reproductive number and sampling proportion. We used a multitype birth-death model in BDMM to quantify the prior probability distribution for the trees. We defined two types that are connected by the same constant type-change (‘migration’) rate. One type is sampled with a constant rate, thus corresponding to our study population. The other type, by contrast, was not sampled and, therefore, includes everything outside of the study population. All samples were assigned the same tip type, corresponding to the type with nonzero sampling rate. At two points in time, April 14, 2021, and July 23, 2023, we allowed the reproductive number and sampling proportion for both types to change. We defined the change points so that the time intervals have a similar length. To test whether we can capture the overall dynamics well with only three intervals, we also analysed the data allowing the reproductive number to change at six points in time. These were February 24, 2021, April 7, 2021, May 19, 2021, June 30, 2021, August 11, 2021, and September 22, 2021. The rate to become noninfectious was fixed to 36.5 year^-1^. We also fixed the geo-frequencies parameter, representing the probability of the root node of the tree being assigned to either type, to 1 for the unsampled and 0 for the sampled type. This induces the assumption that every Pango lineage includes samples from at least two independent introductions, as the root of the lineage is outside of the study population. For the reproductive number, we set a lognormal distribution with mean 0.0 and standard deviation 4.0 as prior for all types and intervals (Supplemental Table S7). As prior probability for the sampling proportion, we chose a Beta(50, 950) distribution. For all other parameters, we kept the default priors. The following steps were then carried out as discussed above. As the sampling period covers the replacement of the variant of concern Alpha by Delta, our data sets include genome sequences from lineages that have shown to differ in their transmissibility. To see whether we can quantify the transmission difference in our study population, we also set up the same analysis as above with variant stratification: We inferred a different reproductive number for the Pango lineage B.1.1.7 (Alpha) and for all included Pango lineages belonging to Delta. We ran all MCMC chains for $3\times 10^{8}$ steps, and when necessary for an additional 10^8^ steps. As the multitype birth-death model allows for many different model variations and can thus be adapted to the setup of interest in each study, we opted to first test the performance of the method on synthetic data simulated under our model. The respective methodology is detailed in Supplemental Text S6.

#### Results and interpretation

**Figure 5.**
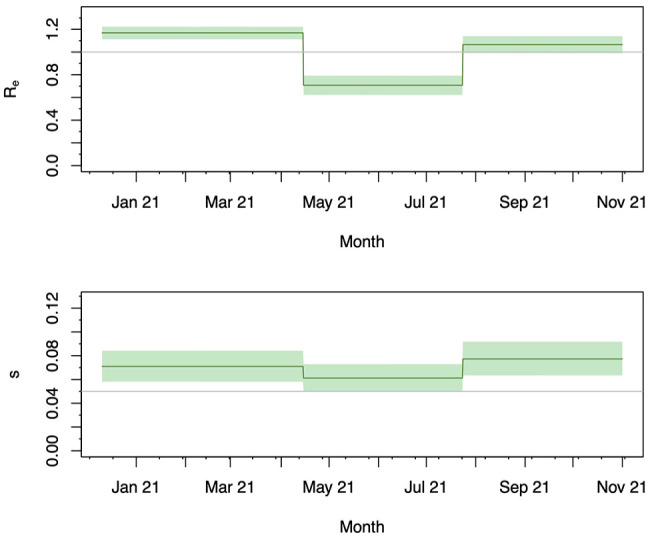
Case study 3 – Spatiotemporal pandemic excerpt: Effective reproductive number (upper panel) and sampling proportion (lower panel) estimated for the sampled study population. Both parameters were allowed to change on April 14 and July 23, 2021. The bold line shows the median posterior estimate and the shaded area the 95% HPDI. The grey line marks an $R_{e}$ of 1.0 (upper panel) and the prior mean of the sampling proportion (lower panel).

In Supplemental Figure S8, we show the results of 50 simulation replicates, 40 out of which reached ESS values above 200 for all estimated parameters. In all of them, the HPDI coverage is high, with a value close to 100%, and the relative bias is small. This suggests that the method can recover the correct transmission dynamics from a single sampled population under our simulation conditions, particularly if it converges well. We thus applied the method to our data set to infer the transmission dynamics in three equally long intervals to quantify temporal changes within the considered region. The resulting estimates of the effective reproductive number ( $R_{e}$ ) and sampling proportion ( s ) are shown in Figure 5. The estimated sampling proportion is close to the expected value of around 5%, as included in the model through an informative prior. Considering the effective reproductive number, we estimate a value above one for the first and third interval, and below one in the second interval. This aligns with the dynamics found on the national level, with decreased transmission in the early summer months that increased over the summer holiday period.^98^ In Supplemental Figure S5, we include a sensitivity analysis in which the effective reproductive number is allowed to change at six equidistant points in time instead of three. This shows that three intervals are sufficient to capture the overall dynamics well. Only during the time between July and September, we observe slight differences between the results. With three intervals, we estimate $R_{e}$ to be above one with high certainty, whereas the additional change points reveal a more complex picture: During July and early August, $R_{e}$ likely rises above one before it drops below one until the end of September. These dynamics could be caused by travel related to summer holidays.^99^ However, because the HPDIs of the posterior estimates overlap, these inferences are associated with higher uncertainty. As many of the identified Pango lineages were very small, we also conducted a sensitivity analysis with a different cluster exclusion threshold. The estimates for $R_{e}$ and s are presented in Supplemental Figure S6, which illustrates that our results are robust to changes in the cluster exclusion threshold. Finally, as the sampling period spans the replacement of the variant of concern Alpha by Delta, we also discuss the sensitivity of our results to stratifying the transmission dynamics per variant. The posterior distribution of $R_{e}$ for each variant is visualised in Supplemental Figure S7. We see that in the first interval we recover estimates with high uncertainty for the Delta variant, as only very few trees span that far back. For Alpha, in contrast, the last interval is characterised by a high HPDI due to the very small number of samples collected during this interval. For the first and last intervals, comparisons are, therefore, not feasible. In the second interval, however, we find the variant-specific $R_{e}$ to be distinctly different. Although the overall dynamics, shown in Figure 5, reconstruct $R_{e}$ to be below one, as is the case for the Alpha variant, the posterior distribution for Delta puts a higher percentage of the probability density on values above one. This illustrates the dynamics of the variant replacement taking place during this time.

This case study shows that without any additional source of information, we are able to disentangle the transmission dynamics of our sampled population and infer the dynamics that shape the diversity outside of this population. Similar approaches based on coalescent tree priors have previously been successfully employed on SARS-CoV-2 data.^84,100,101^ The decrease in the effective reproductive number in the interval between April 14, 2021, and July 23, 2021, can be caused by multiple factors, like nonpharmaceutical interventions implemented locally and increasing temperature.^102,103^ The increase above one in the following interval is most plausibly a result of behavioural changes due to the summer holidays, as reported previously.^99^

## Discussion

With this article, we provide a comprehensive overview of how phylodynamic birth-death models can be used to inform transmission dynamics of SARS-CoV-2. We have summarised all steps that constitute such an analysis and highlighted several situations in which model assumptions and data properties might not align and thus profit from additional validation assessments. To illustrate the versatility of these methods, we have presented and discussed three case studies from the COVID-19 pandemic in Germany. These show how we can disentangle complex epidemiological patterns purely from time-stamped genomic data through Bayesian inference using birth-death-sampling models. In case study 1, we find indications of a superspreading event in the initial phase of the first larger outbreak in Germany by allowing a change in the reproductive number directly after a large-scale gathering. By simultaneously estimating the time-scaled phylodynamic tree we can, in addition, provide a time interval for the date of the first transmission event of the outbreak, based on the observed mutational pattern and estimates of the mutation rate from the literature. Case study 2 takes a comparative approach by focusing on the estimation of the relative difference in the transmission rate between community spread and a nosocomial outbreak. Our results indicate pronounced levels of SARS-CoV-2 transmission within the hospital, emphasising the crucial role of implementing preventive measures effectively within healthcare facilities. In case study 3, we demonstrate the usability of the methods in cases where we do not consider clonal outbreaks. Even in the presence of complex population substructure, we can robustly quantify piecewise constant intervals of the effective reproductive number. The resulting analyses show that the dynamics in the abstractly defined subpopulation well resemble what the dynamics on the national level look like.

Despite their diverse applicability, the discussed methods also come with multiple limitations: First, fundamental challenges may arise from the mathematical properties of the model and simplifying assumptions made within the mathematical models, which may not necessarily be met by the analysed data. The mathematical properties include potential identifiability issues caused by multiple different parameter combinations yielding the same likelihood value and difficulties caused by limited sample size and uncertainty in the tree structure.^22,25^ One of the simplifying assumptions is, for example, the piecewise constant nature of all rate parameters. Particularly for the sampling process, this can lead to pronounced biases if the sampling rate is not actually piecewise constant, but rather changing exponentially through time.^30^ In addition, if the phylogeny is not a good approximation of the transmission tree, for example because the evolutionary and epidemiological processes do not act on similar timescales, this can bias parameter estimates. In that situation, the phylogeny and transmission tree can be modelled separately.^104^ Second, challenges arise from specific properties of the data. For SARS-CoV-2, this entails the large amount of available sequence data, the low genetic diversity and the unknown substructure in these data sets.^105^ The small number of expected substitutions over time scales of weeks or few months and thereby high sequence similarity in data sets sampled over short time periods result in weak phylogenetic signals. In addition, the existing models are computationally expensive due to numerical integration over the high-dimensional tree space. Therefore they cannot easily be applied to large data sets.^106,107^ Recently, methodological advancements, such as PIQMEE^108^ and Thorney BEAST https://beast.community/thorney_beast, last visited May 28, 2024), have enabled phylogenetic analysis in a Bayesian framework of data sets of more than tens of thousands of genomes. Additionally, deep learning approaches have been implemented to estimate epidemiological parameters from phylogenetic trees, allowing fast and accurate inference from large trees.^109^

During the COVID-19 pandemic, it has become particularly important to understand when these comprehensive but complex frameworks are necessary and where simpler methods can yield results of comparable quality. For inference of trees from many genetically very closely related sequences, maximum parsimony approaches have, for example, been shown to perform comparably to more elaborate maximum likelihood or Bayesian analyses while being computationally much less expensive.^110,111^ Although, generally, the least complex method that can provide accurate estimates should be chosen, the assessment of accuracy is not always easy. Simpler models might, for example, be able to estimate the same parameter faster, but lack more detailed quantifications of uncertainty. Bayesian inference, on the contrary, provides a full probabilistic framework that directly quantifies uncertainty in joint probabilities for all parameters of interest. These joint probabilities consider all model components, accounting for correlations between them. Bayesian inference also allows direct model assessment by generating random samples from the generative probability distributions. Such simulations can provide insights into the performance in specific situations. It is important to keep in mind, though, that conclusions from simulation studies are based on synthetic data, which may lack important aspects of real-world data.^112^ Because of the hierarchical structure of Bayesian models, the level of complexity can also be adjusted by making assumptions about parameter values through prior point or range estimates of parameters like the substitution rate or even the phylogenetic tree.^113^ Other data types can also be integrated into the analysis via prior probability distributions, bringing together different lines of evidence.^114^ In addition, especially when choosing a complex Bayesian inference method, the validation of results by simpler methodologies can be considered good practice.

## Conclusions

As we demonstrate here, Bayesian methods can yield valuable information, particularly on well-defined and moderately-sized data sets. In case study 1, we show how we can easily retrace complex seeding dynamics of a large outbreak, that are otherwise only accessible through very time and resource-intensive contact tracing efforts. Case study 2 combines surveillance and outbreak data in a joint analysis setting, thus providing a comparable quantification. Even for data sets for which we cannot precisely define the study population, we are able to disentangle the unknown substructure and estimate the respective transmission dynamics, as shown in case study 3. Nevertheless, these three case studies illustrate only some of the relevant epidemiological information that can be extracted from genome data through phylodynamic models: A multitude of other studies has been investigating aspects such as the origin dates and intrinsic differences in transmissibility of new viral lineages, including variants of concern (see Attwood et al,^38^ Martin et al,^115^ Volz^116^ and references therein).

## Supplemental Material

sj-pdf-1-bbi-10.1177_11779322251321065 – Supplemental material for Evolutionary and epidemic dynamics of COVID-19 in Germany exemplified by three Bayesian phylodynamic case studies Supplemental material, sj-pdf-1-bbi-10.1177_11779322251321065 for Evolutionary and epidemic dynamics of COVID-19 in Germany exemplified by three Bayesian phylodynamic case studies by Sanni Översti, Ariane Weber, Viktor Baran, Bärbel Kieninger, Alexander Dilthey, Torsten Houwaart, Andreas Walker, Wulf Schneider-Brachert and Denise Kühnert in Bioinformatics and Biology Insights

## References

[1] Balloux F Brønstad Brynildsrud O van Dorp L , et al. From theory to practice: translating whole-genome sequencing (WGS) into the clinic. Trends Microbiol. 2018;26:1035-1048. doi:10.1016/j.tim.2018.08.004 30193960 PMC6249990

[2] Gardy JL Loman NJ. Towards a genomics-informed, real-time, global pathogen surveillance system. Nat Rev Genet. 2018;19:9-20. doi:10.1038/nrg.2017.88 29129921 PMC7097748

[3] Armstrong GL MacCannell DR Taylor J , et al. Pathogen genomics in public health. N Engl J Med. 2019;381:2569-2580. doi:10.1056/NEJMsr1813907 31881145 PMC7008580

[4] van Dorp L Houldcroft CJ Richard D Balloux F. COVID-19, the first pandemic in the post-genomic era. Curr Opin Virol. 2021;50:40-48. doi:10.1016/j.coviro.2021.07.002 34352474 PMC8275481

[5] Hill V Ruis C Bajaj S Pybus OG Kraemer MUG . Progress and challenges in virus genomic epidemiology. Trends Parasitol. 2021;37:1038-1049. doi:10.1016/j.pt.2021.08.007 34620561

[6] Oh DY Hölzer M Paraskevopoulou S , et al. Advancing precision vaccinology by molecular and genomic surveillance of severe acute respiratory syndrome coronavirus 2 in Germany, 2021. Clin Infect Dis. 2022;75(Suppl. 1):s110-s120. doi:10.1093/cid/ciac399 PMC9278222 35749674

[7] Stockdale JE Liu P Colijn C. The potential of genomics for infectious disease forecasting. Nat Microbiol. 2022;7:1736-1743. doi:10.1038/s41564-022-01233-6 36266338

[8] Grenfell BT Pybus OG Gog JR , et al. Unifying the epidemiological and evolutionary dynamics of pathogens. Science. 2004;303:327-332. doi:10.1126/science.1090727 14726583

[9] Baele G Suchard MA Rambaut A Lemey P. Emerging concepts of data integration in pathogen phylodynamics. Syst Biol. 2017;66:e47-e65. doi:10.1093/sysbio/syw054 PMC5837209 28173504

[10] Rife BD Mavian C Chen X , et al. Phylodynamic applications in 21st century global infectious disease research. Glob Health Res Policy. 2017;2:13. doi:10.1186/s41256-017-0034-y 29202081 PMC5683535

[11] McElreath R. Statistical Rethinking: A Bayesian Course with Examples in R and Stan. 2nd ed. Chapman and Hall/CRC;2020: doi:10.1201/9780429029608

[12] Drummond AJ Bouckaert RR. Bayesian Evolutionary Analysis with BEAST. Cambridge University Press; 2015.

[13] Vegvari C Abbott S Ball F , et al. Commentary on the use of the reproduction number R during the COVID-19 pandemic. Stat Methods Med Res. 2022;31:1675-1685. doi:10.1177/09622802211037079 34569883 PMC9277711

[14] Gostic KM McGough L Baskerville EB , et al. Practical considerations for measuring the effective reproductive number, Rt. PLoS Comput Biol. 2020;16:e1008409. doi:10.1371/journal.pcbi.1008409 PMC7728287 33301457

[15] Nascimento FF Reis MD Yang Z. A biologist’s guide to Bayesian phylogenetic analysis. Nat Ecol Evol. 2017;1:1446-1454. doi:10.1038/s41559-017-0280-x 28983516 PMC5624502

[16] Bouckaert R Vaughan TG Barido-Sottani J , et al. BEAST 2.5: An advanced software platform for Bayesian evolutionary analysis. PLoS Comput Biol. 2019;15:e1006650. doi:10.1371/journal.pcbi.1006650 PMC6472827 30958812

[17] Kendall DG. On the generalized ‘birth-and-death’ process. Ann Math Stat. 1948;19:1-15. doi:10.1214/aoms/1177730285.

[18] MacPherson A Louca S McLaughlin A Joy JB Pennell MW. Unifying phylogenetic birth-death models in epidemiology and macroevolution. Syst Biol. 2022;71:172-189. doi:10.1093/sysbio/syab049 PMC8972974 34165577

[19] Kingman JFC . The coalescent. Stoch Process Their Appl. 1982;13:235-248. doi:10.1016/0304-4149(82)90011-4

[20] Featherstone LA Zhang JM Vaughan TG Duchene S. Epidemiological inference from pathogen genomes: a review of phylodynamic models and applications. Virus Evol. 2022;8:veac045. doi:10.1093/ve/veac045 PMC9241095 35775026

[21] Gavryushkina A Welch D Stadler T Drummond AJ. Bayesian inference of sampled ancestor trees for epidemiology and fossil calibration. PLoS Comput Biol. 2014;10:e1003919. doi:10.1371/journal.pcbi.1003919 PMC4263412 25474353

[22] Louca S McLaughlin A MacPherson A Joy JB Pennell MW. Fundamental identifiability limits in molecular epidemiology. Mol Biol Evol. 2021;38:4010-4024. doi:10.1093/molbev/msab149 34009339 PMC8382926

[23] Legried B Terhorst J. A class of identifiable phylogenetic birth-death models. Proc Natl Acad Sci. 2022;119:e2119513119. doi:10.1073/pnas.2119513119 PMC9436344 35994663

[24] Truman K Vaughan TG Gavryushkin A Gavryushkina A. The fossilised birth-death model is identifiable. Syst Biol. 2024;74: 112-123. doi:10.1093/sysbio/syae058 PMC11997801 39436077

[25] Legried B Terhorst J. Identifiability and inference of phylogenetic birth-death models. J Theor Biol. 2023;568:111520. doi:10.1016/j.jtbi.2023.111520 37148965

[26] Morlon H Robin S Hartig F. Studying speciation and extinction dynamics from phylogenies: addressing identifiability issues. Trends Ecol Evol. 2022;37:497-506. doi:10.1016/j.tree.2022.02.004 35246322

[27] Kopperud BT Magee AF Höhna S. Rapidly changing speciation and extinction rates can be inferred in spite of nonidentifiability. Proc Natl Acad Sci. 2023;120:e2208851120. doi:10.1073/pnas.2208851120 PMC9963352 36757894

[28] Boskova V Bonhoeffer S Stadler T. Inference of epidemiological dynamics based on simulated phylogenies using birth-death and coalescent models. PLoS Comput Biol. 2014;10:e1003913. doi:10.1371/journal.pcbi.1003913 PMC4222655 25375100

[29] Grubaugh ND Ladner JT Lemey P , et al. Tracking virus outbreaks in the twenty-first century. Nat Microbiol. 2018;4:10-19. doi:10.1038/s41564-018-0296-2 30546099 PMC6345516

[30] Volz EM Frost SDW . Sampling through time and phylodynamic inference with coalescent and birth-death models. J R Soc Interface. 2014;11:20140945. doi:10.1098/rsif.2014.0945 25401173 PMC4223917

[31] Inward RPD Parag KV Faria NR . Using multiple sampling strategies to estimate SARS-CoV-2 epidemiological parameters from genomic sequencing data. Nat Commun. 2022;13:5587. doi:10.1038/s41467-022-32812-0 36151084 PMC9508174

[32] Stadler T Kühnert D Bonhoeffer S Drummond AJ. Birth-death skyline plot reveals temporal changes of epidemic spread in HIV and hepatitis C virus (HCV). Proc Natl Acad Sci U S A. 2013;110:228-233. doi:10.1073/pnas.1207965110 23248286 PMC3538216

[33] Aggarwal D Warne B Jahun AS , et al. Genomic epidemiology of SARS-CoV-2 in a UK university identifies dynamics of transmission. Nat Commun. 2022;13:751. doi:10.1038/s41467-021-27942-w 35136068 PMC8826310

[34] Vaughan TG Scire J Nadeau SA Stadler T. Estimates of early outbreak-specific SARS-CoV-2 epidemiological parameters from genomic data. Proc Natl Acad Sci. 2024;121:e2308125121. doi:10.1073/pnas.2308125121 PMC10786264 38175864

[35] Weber A Översti S Kühnert D. Reconstructing relative transmission rates in Bayesian phylodynamics: two-fold transmission advantage of Omicron in Berlin, Germany during December 2021. Virus Evol. 2023;9:vead070. doi:10.1093/ve/vead070 PMC10725310 38107332

[36] Kühnert D Stadler T Vaughan TG Drummond AJ. Phylodynamics with migration: a computational framework to quantify population structure from genomic data. Mol Biol Evol. 2016;33:2102-2116. doi:10.1093/molbev/msw064 27189573 PMC4948704

[37] Barido-Sottani J Schwery O Warnock RCM Zhang C Wright AM. Practical guidelines for Bayesian phylogenetic inference using Markov Chain Monte Carlo (MCMC). Open Res Eur. 2023;3:204. doi:10.12688/openreseurope.16679.1 38481771 PMC10933576

[38] Attwood SW Hill SC Aanensen DM Connor TR Pybus OG. Phylogenetic and phylodynamic approaches to understanding and combating the early SARS-CoV-2 pandemic. Nat Rev Genet. 2022;23:547-562. doi:10.1038/s41576-022-00483-8 35459859 PMC9028907

[39] Rich SN Richards V Mavian C , et al. Application of phylodynamic tools to inform the public health response to COVID-19: qualitative analysis of expert opinions. JMIR Form Res. 2023;7:e39409. doi:10.2196/39409 PMC10131930 36848460

[40] Möller S du Plessis L Stadler T. Impact of the tree prior on estimating clock rates during epidemic outbreaks. Proc Natl Acad Sci U S A. 2018;115:4200-4205. doi:10.1073/pnas.1713314115 29610334 PMC5910814

[41] Walker A Houwaart T Wienemann T , et al. Genetic structure of SARS-CoV-2 reflects clonal superspreading and multiple independent introduction events, North-Rhine Westphalia, Germany, February and March 2020. Euro Surveill. 2020;25:2000746. doi:10.2807/1560-7917.ES.2020.25.22.2000746 32524946 PMC7336109

[42] Walker A Houwaart T Finzer P , et al. Characterization of severe acute respiratory syndrome coronavirus 2 (SARS-CoV-2) Infection clusters based on integrated genomic surveillance, outbreak analysis and contact tracing in an urban setting. Clin Infect Dis. 2022;74:1039-1046. doi:10.1093/cid/ciab588 34181711 PMC8406867

[43] Elbe S Buckland-Merrett G. Data, disease and diplomacy: GISAID’s innovative contribution to global health. Glob Chall. 2017;1:33-46. doi:10.1002/gch2.1018 31565258 PMC6607375

[44] Shu Y McCauley J. GISAID: Global initiative on sharing all influenza data – from vision to reality. Euro Surveill. 2017;22:30494. doi:10.2807/1560-7917.ES.2017.22.13.30494 28382917 PMC5388101

[45] Khare S Gurry C Freitas L , et al. GISAID’s role in pandemic response. China CDC Wkly. 2021;3:1049-1051. doi:10.46234/ccdcw2021.255 34934514 PMC8668406

[46] National Center for Biotechnology Information. National Center for Biotechnology Information (NCBI) [Internet]. National Library of Medicine, National Center for Biotechnology Information;1988. Accessed May 16, 2024. https://www.ncbi.nlm.nih.gov/

[47] Lemmon AR Brown JM Stanger-Hall K Lemmon EM. The effect of ambiguous data on phylogenetic estimates obtained by maximum likelihood and Bayesian inference. Syst Biol. 2009;58:130-145. doi:10.1093/sysbio/syp017 20525573 PMC7539334

[48] Katoh K Standley DM. MAFFT multiple sequence alignment software version 7: improvements in performance and usability. Mol Biol Evol. 2013;30:772-780. doi:10.1093/molbev/mst010 23329690 PMC3603318

[49] Pervez MT Babar ME Nadeem A , et al. Evaluating the accuracy and efficiency of multiple sequence alignment methods. Evol Bioinform Online. 2014;10:205-217. doi:10.4137/EBO.S19199 25574120 PMC4267518

[50] Ogden TH Rosenberg MS. Multiple sequence alignment accuracy and phylogenetic inference. Syst Biol. 2006;55:314-328. doi:10.1080/10635150500541730. 16611602

[51] Alqahtani A Almutairy M. Evaluating the performance of multiple sequence alignment programs with application to genotyping SARS-CoV-2 in the Saudi population. Computation. 2023;11:212. doi:10.3390/computation11110212.

[52] Waterhouse AM Procter JB Martin DMA Clamp M Barton GJ. Jalview Version 2 – a multiple sequence alignment editor and analysis workbench. Bioinformatics. 2009;25:1189-1191. doi:10.1093/bioinformatics/btp033 19151095 PMC2672624

[53] De Maio N Walker C Borger R Weilguny L Slodkowicz G Goldman N . Masking strategies for SARS-CoV-2 alignments – SARS-CoV-2 coronavirus / Software and Tools. Virological. July 24, 2020. Accessed May 16, 2024. https://virological.org/t/masking-strategies-for-sars-cov-2-alignments/480

[54] Turakhia Y De Maio N Thornlow B , et al. Stability of SARS-CoV-2 phylogenies. PLoS Genet. 2020;16:e1009175. doi:10.1371/journal.pgen.1009175 PMC7721162 33206635

[55] Rambaut A Holmes EC O’Toole Á , et al. A dynamic nomenclature proposal for SARS-CoV-2 lineages to assist genomic epidemiology. Nat Microbiol. 2020;5:1403-1407. doi:10.1038/s41564-020-0770-5 32669681 PMC7610519

[56] Rambaut A Lam TT Max Carvalho L Pybus OG. Exploring the temporal structure of heterochronous sequences using TempEst (formerly Path-O-Gen). Virus Evol. 2016;2:1-7. doi:10.1093/ve/vew007 PMC4989882 27774300

[57] Byrne AW McEvoy D Collins AB , et al. Inferred duration of infectious period of SARS-CoV-2: rapid scoping review and analysis of available evidence for asymptomatic and symptomatic COVID-19 cases. BMJ Open. 2020;10:e039856. doi:10.1136/bmjopen-2020-039856 PMC7409948 32759252

[58] Lam A Duchene S. The impacts of low diversity sequence data on phylodynamic inference during an emerging epidemic. Viruses. 2021;13:79. doi:10.3390/v13010079 33430050 PMC7826997

[59] Nadeau SA Vaughan TG Scire J Huisman JS Stadler T. The origin and early spread of SARS-CoV-2 in Europe. Proc Natl Acad Sci U S A. 2021;118:1-8. doi:10.1073/pnas.2012008118 PMC7936359 33571105

[60] Hill V Du Plessis L Peacock TP , et al. The origins and molecular evolution of SARS-CoV-2 lineage B.1.1.7 in the UK. Virus Evol. 2022;8:veac080. doi:10.1093/ve/veac080 PMC9752794 36533153

[61] Siedner MJ Boucau J Gilbert RF , et al. Duration of viral shedding and culture positivity with postvaccination SARS-CoV-2 delta variant infections. JCI Insight. 2022;7:e155483. doi:10.1172/jci.insight.155483 10.1172/jci.insight.155483 PMC8855795 34871181

[62] NIID. Active Epidemiological Investigation on SARS-CoV-2 Infection Caused by Omicron Variant (Pango Lineage B.1.1.529) in Japan: Preliminary Report on Infectious Period. National Institute of Infectious Diseases Disease Control and Prevention Center, National Center for Global Health and Medicine; 2022. Accessed May 16, 2024. https://www.niid.go.jp/niid/en/2019-ncov-e/10884-covid19-66-en.html

[63] Bromham L Duchêne S Hua X Ritchie AM Duchêne DA Ho SYW . Bayesian molecular dating: opening up the black box. Biol Rev Camb Philos Soc. 2018;93:1165-1191. doi:10.1111/brv.12390 29243391

[64] Bromham L. Six impossible things before breakfast: assumptions, models, and belief in molecular dating. Trends Ecol Evol. 2019;34:474-486. doi:10.1016/j.tree.2019.01.017 30904189

[65] Posada D Crandall KA. Selecting the best-fit model of nucleotide substitution. Syst Biol. 2001;50:580-601. doi:10.1080/106351501750435121 12116655

[66] Darriba D Taboada GL Doallo R Posada D. JModelTest 2: more models, new heuristics and parallel computing. Nat Methods. 2012;9:772. doi:10.1038/nmeth.2109 PMC4594756 22847109

[67] Lanfear R Calcott B Ho SY Guindon S. PartitionFinder: combined selection of partitioning schemes and substitution models for phylogenetic analyses. Mol Biol Evol. 2012;29:1695-1701. doi:10.1093/molbev/mss020 22319168

[68] Bouckaert RR Drummond AJ. bModelTest: Bayesian phylogenetic site model averaging and model comparison. BMC Evol Biol. 2017;17:1-11. doi:10.1186/s12862-017-0890-6 28166715 PMC5294809

[69] Hasegawa M Kishino H Yano T. Dating of the human-ape splitting by a molecular clock of mitochondrial DNA. J Mol Evol. 1985;22:160-174. doi:10.1007/BF02101694 3934395

[70] Yang Z. Maximum likelihood phylogenetic estimation from DNA sequences with variable rates over sites: approximate methods. J Mol Evol. 1994;39:306-314. doi:10.1007/BF00160154 7932792

[71] Duchene S Featherstone L Haritopoulou-Sinanidou M Rambaut A Lemey P Baele G. Temporal signal and the phylodynamic threshold of SARS-CoV-2. Virus Evol. 2020;6:veaa061. doi:10.1093/ve/veaa061 PMC7454936 33235813

[72] Nadeau SA Vaughan TG Beckmann C , et al. Swiss public health measures associated with reduced SARS-CoV-2 transmission using genome data. Sci Transl Med. 2023;15:eabn7979. 10.1126/scitranslmed.abn7979 PMC9765449 36346321

[73] Ghafari M Du Plessis L Pybus OG Katzourakis A . Time dependence of SARS-CoV-2 substitution rates. Virological. October 27, 2020. Accessed May 16, 2024. https://virological.org/t/time-dependence-of-sars-cov-2-substitution-rates/542

[74] Rambaut A Drummond AJ Xie D Baele G Suchard MA. Posterior summarization in Bayesian phylogenetics using Tracer 1.7. Syst Biol. 2018;67:901-904. doi:10.1093/sysbio/syy032 29718447 PMC6101584

[75] RStudio team. RStudio: Integrated Development for R, 2020. Accessed May 16, 2024. http://www.rstudio.com/

[76] Paradis E Schliep K. ape 5.0: an environment for modern phylogenetics and evolutionary analyses in R. Bioinformatics. 2019;35:526-528. doi:10.1093/bioinformatics/bty633 30016406

[77] Yu G Smith DK Zhu H Guan Y Lam TTY . ggtree: an r package for visualization and annotation of phylogenetic trees with their covariates and other associated data. Methods Ecol Evol. 2017;8:28-36. doi:10.1111/2041-210X.12628

[78] Wickham H . ggplot2: Elegant Graphics for Data Analysis. Springer;2016. doi:10.1007/978-3-319-24277-4.

[79] Finnesgard E . utile.visuals: Create visuals for publication, 2023. Accessed May 16, 2024. https://efinite.github.io/utile.visuals/

[80] Streeck H Schulte B Kümmerer BM , et al. Infection fatality rate of SARS-CoV2 in a super-spreading event in Germany. Nat Commun. 2020;11:1-12. doi:10.1038/s41467-020-19509-y 33203887 PMC7672059

[81] Robert Koch-Institut. Coronavirus Disease 2019 (COVID-19) Daily Situation Report 05/03/2020. RKI; Mar 2020. Accessed May 16, 2024. https://www.rki.de/DE/Content/InfAZ/N/Neuartiges_Coronavirus/Situationsberichte/2020-03-05-en.pdf?__blob=publicationFile

[82] Korencak M Sivalingam S Sahu A , et al. Reconstruction of the origin of the first major SARS-CoV-2 outbreak in Germany. Comput Struct Biotechnol J. 2022;20:2292-2296. doi:10.1016/j.csbj.2022.05.011 35574268 PMC9088089

[83] Volz EM Siveroni I. Bayesian phylodynamic inference with complex models. PLoS Comput Biol. 2018;14:e1006546. doi:10.1371/JOURNAL.PCBI.1006546 PMC6258546 30422979

[84] Miller D Martin MA Harel N , et al. Full genome viral sequences inform patterns of SARS-CoV-2 spread into and within Israel. Nat Commun. 2020;11:5518. doi:10.1038/s41467-020-19248-0 33139704 PMC7606475

[85] Meehan MT Hughes A Ragonnet RR , et al. Replicating superspreader dynamics with compartmental models. Sci Rep. 2023;13:15319. doi:10.1038/s41598-023-42567-3 37714942 PMC10504364

[86] Robert Koch-Institut. SARS-CoV-2 Infektionen in Deutschland. Published May 13, 2024. doi:10.5281/zenodo.11183325

[87] Liu Y Gayle AA Wilder-Smith A Rocklöv J. The reproductive number of COVID-19 is higher compared to SARS coronavirus. J Travel Med. 2020;27:1-4. doi:10.1093/jtm/taaa021 PMC7074654 32052846

[88] Park SW Bolker BM Champredon D , et al. Reconciling early-outbreak estimates of the basic reproductive number and its uncertainty: framework and applications to the novel coronavirus (SARS-CoV-2) outbreak. J R Soc Interface. 2020;17:20200144. doi:10.1098/rsif.2020.0144 32693748 PMC7423425

[89] Prada JP Maag LE Siegmund L , et al. Estimation of R0 for the spread of SARS-CoV-2 in Germany from excess mortality. Sci Rep. 2022;12:17221. doi:10.1038/s41598-022-22101-7 36241688 PMC9562071

[90] Bouckaert RR. DensiTree: making sense of sets of phylogenetic trees. Bioinformatics. 2010;26:1372-1373. doi:10.1093/bioinformatics/btq110 20228129

[91] Cooper BS Evans S Jafari Y , et al. The burden and dynamics of hospital-acquired SARS-CoV-2 in England. Nature. 2023;623:132-138. doi:10.1038/s41586-023-06634-z 37853126 PMC10620085

[92] Abbas M Robalo Nunes T Martischang R , et al. Nosocomial transmission and outbreaks of coronavirus disease 2019: the need to protect both patients and healthcare workers. Antimicrob Resist Infect Control. 2021;10:7. doi:10.1186/s13756-020-00875-7 33407833 PMC7787623

[93] Shirreff G Zahar JR Cauchemez S Temime L Opatowski L , EMEA-MESuRS Working Group on the Nosocomial Modelling of SARS-CoV-22. Measuring basic reproduction number to assess effects of nonpharmaceutical interventions on nosocomial SARS-CoV-2 transmission. Emerg Infect Dis. 2022;28:1345-1354. doi:10.3201/eid2807.212339 35580960 PMC9239897

[94] Thompson HA Mousa A Dighe A , et al. Severe acute respiratory syndrome coronavirus 2 (SARS-CoV-2) setting-specific transmission rates: a systematic review and meta-analysis. Clin Infect Dis. 2021;73:e754-e764. doi:10.1093/cid/ciab100 PMC7929012 33560412

[95] Stockdale JE Anderson SC Edwards AM , et al. Quantifying transmissibility of SARS-CoV-2 and impact of intervention within long-term healthcare facilities. R Soc Open Sci. 2022;9:211710. doi:10.1098/rsos.211710 35242355 PMC8753163

[96] Rivett L Sridhar S Sparkes D , et al. Screening of healthcare workers for SARS-CoV-2 highlights the role of asymptomatic carriage in COVID-19 transmission. eLife. 2020;9:e58728. doi:10.7554/eLife.58728 PMC7314537 32392129

[97] O’Toole Á Scher E Underwood A , et al. Assignment of epidemiological lineages in an emerging pandemic using the pangolin tool. Virus Evol. 2021;7:veab064. doi:10.1093/ve/veab064 PMC8344591 34527285

[98] Tolksdorf K Loenenbach A Buda S. Dritte Aktualisierung der ‘Retrospektiven Phaseneinteilung der COVID-19-Pandemie in Deutschland’. Published September 22, 2022. Accessed May 16, 2024. https://edoc.rki.de/handle/176904/10260

[99] Plümper T Neumayer E. Fueling the Covid-19 pandemic: summer school holidays and incidence rates in German districts. J Public Health. 2021;43:e415-e422. doi:10.1093/pubmed/fdab080 PMC8083696 33765149

[100] Ragonnet-Cronin M Boyd O Geidelberg L , et al. Genetic evidence for the association between COVID-19 epidemic severity and timing of non-pharmaceutical interventions. Nat Commun. 2021;12:2188. doi:10.1038/s41467-021-22366-y 33846321 PMC8041850

[101] Didelot X Helekal D Kendall M Ribeca P. Distinguishing imported cases from locally acquired cases within a geographically limited genomic sample of an infectious disease. Bioinformatics. 2023;39:btac761. doi:10.1093/bioinformatics/btac761 PMC9805578 36440957

[102] Ganslmeier M Furceri D Ostry JD. The impact of weather on COVID-19 pandemic. Sci Rep. 2021;11:22027. doi:10.1038/s41598-021-01189-3 PMC8585954 34764317

[103] Han J Yin J Wu X Wang D Li C. Environment and COVID-19 incidence: a critical review. J Environ Sci. 2023;124:933-951. doi:10.1016/j.jes.2022.02.016 PMC8858699 36182196

[104] Ypma RJ van Ballegooijen WM Wallinga J. Relating phylogenetic trees to transmission trees of infectious disease outbreaks. Genetics. 2013;195:1055-1062. doi:10.1534/genetics.113.154856 24037268 PMC3813836

[105] Morel B Barbera P Czech L , et al. Phylogenetic analysis of SARS-CoV-2 data is difficult. Mol Biol Evol. 2021;38:1777-1791. doi:10.1093/molbev/msaa314 33316067 PMC7798910

[106] Frost SD Pybus OG Gog JR Viboud C Bonhoeffer S Bedford T. Eight challenges in phylodynamic inference. Epidemics. 2015;10:88-92. doi:10.1016/j.epidem.2014.09.001 25843391 PMC4383806

[107] Cappello L Kim J Liu S Palacios JA. Statistical challenges in tracking the evolution of SARS-CoV-2. Stat Sci. 2022;37:162-182. doi:10.1214/22-sts853 36034090 PMC9409356

[108] Boskova V Stadler T. PIQMEE: Bayesian phylodynamic method for analysis of large data sets with duplicate sequences. Mol Biol Evol. 2020;37:3061-3075. doi:10.1093/molbev/msaa136 32492139 PMC7530608

[109] Voznica J Zhukova A Boskova V , et al. Deep learning from phylogenies to uncover the epidemiological dynamics of outbreaks. Nat Commun. 2022;13:3896. doi:10.1038/s41467-022-31511-0 35794110 PMC9258765

[110] Turakhia Y Thornlow B Hinrichs AS , et al. Ultrafast Sample placement on Existing tRees (UShER) enables real-time phylogenetics for the SARS-CoV-2 pandemic. Nat Genet. 2021;53:809-816. doi:10.1038/s41588-021-00862-7 33972780 PMC9248294

[111] Kramer AM Thornlow B Ye C , et al. Online phylogenetics with matOptimize Produces equivalent trees and is dramatically more efficient for large SARS-CoV-2 phylogenies than de novo and maximum-likelihood implementations. Syst Biol. 2023;72:1039-1051. doi:10.1093/sysbio/syad031 37232476 PMC10627557

[112] Trost J Haag J Höhler D Jacob L Stamatakis A Boussau B. Simulations of sequence evolution: how (un)realistic they are and why. Mol Biol Evol. 2024;41:msad277. doi:10.1093/molbev/msad277 PMC10768886 38124381

[113] Höhna S Hsiang AY. Sequential Bayesian phylogenetic inference. Syst Biol. 2024;73:704-721. doi:10.1093/sysbio/syae020. 38771253

[114] Hassler GW Magee A Zhang Z , et al. Data integration in Bayesian phylogenetics. Annu Rev Stat Appl. 2023;10:353-377. doi:10.1146/annurev-statistics-033021-112532 38774036 PMC11108065

[115] Martin DP Weaver S Tegally H , et al. The emergence and ongoing convergent evolution of the SARS-CoV-2 N501Y lineages. Cell. 2021;184:5189-5200.e7. doi:10.1016/j.cell.2021.09.003 PMC8421097 34537136

[116] Volz E. Fitness, growth and transmissibility of SARS-CoV-2 genetic variants. Nat Rev Genet. 2023;24:724-734. doi:10.1038/s41576-023-00610-z 37328556

