# Supplementary material for: Evolutionary and epidemic dynamics of COVID-19 in Germany exemplified by three Bayesian phylodynamic case studies
Source: Bioinform Biol Insights. 2025 Mar 12;19:11779322251321065. doi: 10.1177/11779322251321065 (PMC11898094; doi:10.1177/11779322251321065)
Supplement: sj-pdf-1-bbi-10.1177_11779322251321065 – Supplemental material for Evolutionary and epidemic dynamics of COVID-19 in Germany exemplified by three Bayesian phylodynamic case studies [file sj-pdf-1-bbi-10.1177_11779322251321065.pdf]

Evolutionary and epidemic dynamics of COVID-19 in Germany exemplified by three Bayesian phylodynamic case studies

Sanni Översti*, Ariane Weber*, Viktor Baran, Bärbel Kieninger, Alexander Dilthey, Torsten Houwaart, Andreas Walker, Wulf Schneider-Brachert, Denise Kühnert

*Contributed equally

Supplementary material

Content

Abbreviations and mathematical symbols

Case study 1 - Superspreading

1. Main analysis: Phylodynamic setup and additional results
   Supplementary table S1 - Priors
   Supplementary figure S1 - MCC tree B.1
2. Sensitivity analyses: Summary statistics
   Supplementary table S2 - Results
3. Simulations: Phylodynamic setup and summary statistics
   Supplementary text S1 - Setup
   Supplementary table S3 - Results

Case study 2 - Hospital outbreak

1. Main analysis: Phylodynamic setup and additional results
   Supplementary text S2 - Setup
   Supplementary figure S2 - Number of sequenced surveillance samples in Düsseldorf between weeks 33 and 51 in 2020
   Supplementary table S4 - Priors
   Supplementary figure S3 - MCC tree for the hospital cluster
2. Sensitivity analyses: Summary statistics
   Supplementary text S3 - Setup
   Supplementary table S5 - Results
3. Simulations: Phylodynamic setup and summary statistics
   Supplementary text S4 - Setup
   Supplementary table S6 - Results

Case study 3 - Spatiotemporal pandemic excerpt

1. Data: Generation and metadata
   Supplementary text S5 - Data generation
   Supplementary figure S4 - Sequence and diagnosis counts

2. Main analysis: Phylodynamic setup
   Supplementary table S7 - Priors
3. Sensitivity analyses: Summary statistics
   Supplementary figure S5 - Sensitivity analysis 1
   Supplementary figure S6 - Sensitivity analysis 2
   Supplementary figure S7 - Sensitivity analysis 3
4. Simulations: Phylodynamic setup and summary statistics
   Supplementary text S6 - Setup
   Supplementary figure S8 - Results

Abbreviations and mathematical symbols

COVID-19	Coronavirus disease 2019
ESS	Effective sample size
HKY85	Hasegawa-Kishino-Yano-85 substitution model
HPDI	Highest posterior density interval
MCC	Maximum clade credibility
MCMC	Markov Chain Monte Carlo
MRCA	Most Recent Common Ancestor
MSA	Multiple sequence alignment
SARS-CoV-2	Severe acute respiratory syndrome coronavirus type 2
UCLD	Uncorrelated log-normal relaxed molecular clock
R	Reproductive number
R_{base}	Baseline effective reproductive number (BDSKYλ model)
R_e	Effective reproductive number
r_λ	Transmission rate ratio (BDSKYλ model)
s	Sampling proportion
Γ	Gamma distribution
δ	Rate to become non-infectious
λ	Transmission rate
μ	Recovery rate
ψ	Sampling rate

Case study 1 - Superspreading

1. Main analysis: Phylodynamic setup and additional results

Below we provide a table summarising all prior probability distributions on model parameters.

Supplementary table S1. Case study 1 - Superspreading: Prior distributions for all analyses. We fixed the rate to become non-infectious to 36.5 y^{-1} and the clock rate to 0.0008 substitutions/site/year, if not otherwise indicated. For κ, gamma shape s, the base frequencies and, where applicable, the UCLD standard deviation we applied the default prior distributions.

Parameter	Main analysis	Sensitivity analysis 1 (Lower coverage incl.)	Sensitivity analysis 2 (Uniform prior on s)	Sensitivity analysis 3 (UCLD relaxed clock)	Sensitivity analysis 4 (Narrow prior on strict clock rate)	Sensitivity analysis 5 (Narrow clock rate prior + R change after root)
$R_{e,1}$	LN(0.0, 4.0)	LN(0.0, 4.0)	LN(0.0, 4.0)	LN(0.0, 4.0)	LN(0.0, 4.0)	LN(0.0, 4.0)
$R_{e,2}$	LN(0.0, 4.0)	LN(0.0, 4.0)	LN(0.0, 4.0)	LN(0.0, 4.0)	LN(0.0, 4.0)	LN(0.0, 4.0)
s	Beta(32.0, 968.0)	Beta(71.0, 929.0)	Unif(0,1)	Beta(32.0, 968.0)	Beta(32.0, 968.0)	Beta(32.0, 968.0)
κ	LN(1.0,1.25)	LN(1.0,1.25)	LN(1.0,1.25)	LN(1.0,1.25)	LN(1.0,1.25)	LN(1.0,1.25)
gamma shape s	Exp(1.0)	Exp(1.0)	Exp(1.0)	Exp(1.0)	Exp(1.0)	Exp(1.0)
Base frequencies	Unif(0.0,1.0)	Unif(0.0,1.0)	Unif(0.0,1.0)	Unif(0.0,1.0)	Unif(0.0,1.0)	Unif(0.0,1.0)
Substitution rate					Exp(8E-4)	Exp(8E-4)
UCLD standard deviation	-	-	-	-	Gamma (0.5396, 0.3819)	-

The additional results below show the posterior summary of the phylodynamic tree sample of sequences belonging to the Pango lineage B.1.

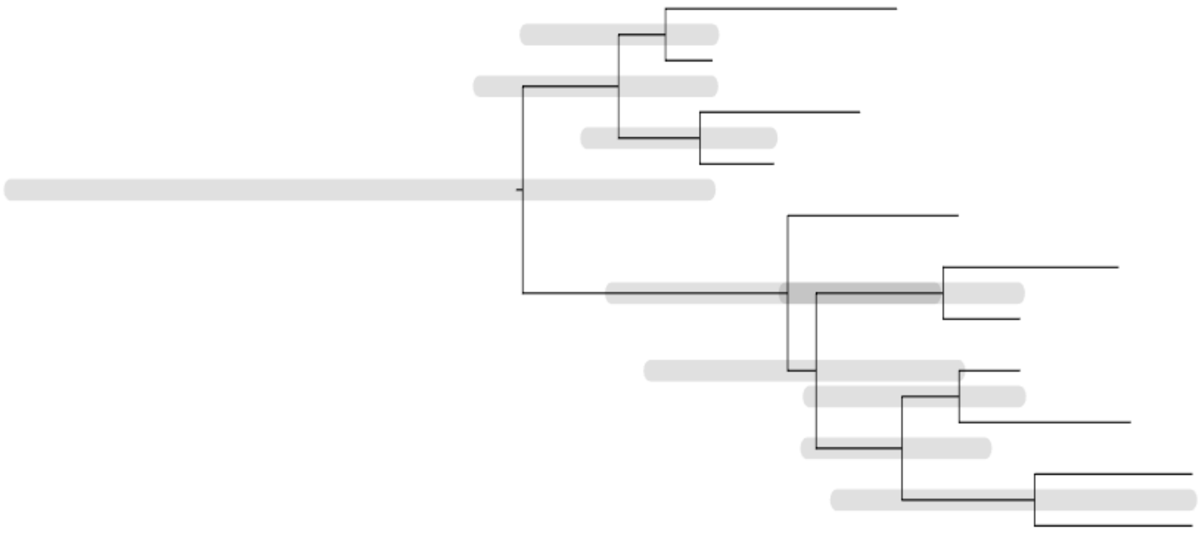

Supplementary figure S1. Case study 1 - Superspreading: Summary (maximum clade credibility with median heights) tree for Pango lineage B.1. Grey horizontal bars show the 95% HPDI for the internal node heights. The estimated 95% HPDI for the root node is [0.107, 0.262].

2. Sensitivity analyses: Summary statistics

As detailed in the main text, we conducted several sensitivity analyses to demonstrate the behaviour of our posterior estimates if specific properties of the data and model assumptions are changed. In the following we provide an overview table of the posterior estimates of all analyses, the respective prior distributions are shown in Supplementary table S1.

Supplementary table S2. Case study 1 - Superspreading: Sensitivity analyses - Inferred posterior. Median and 95% HPDIs for posterior estimates of the main and two sensitivity analyses. Values in italics were fixed during the analysis and not estimated. 1) Including all genomes with less than 5% of 'N's, 2) with a uniform prior on the sampling proportion, 3) using a UCLD relaxed clock model with fixed mean rate, 4) estimating the mean rate with a narrow prior for a strict clock model and 5) as 4) but with the change in R_e set to happen 10 days after the root node instead of 16 February 2020.

Parameter	Main analysis	Sensitivity analysis 1	Sensitivity analysis 2	Sensitivity analysis 3	Sensitivity analysis 4	Sensitivity analysis 5
$R_{e,1}$	12.35<br>[2.86, 51.6]	15.48<br>[4.81, 41.5]	4.50<br>[0.0, 25.9]	4.80<br>[1.20, 14.20]	1.50<br>[1.00, 3.20]	11.90<br>[6.70, 19.10]
$R_{e,1}$	0.92<br>[0.35, 1.55]	0.78<br>[0.61,0.95]	0.77<br>[0.41, 1.19]	0.70<br>[0.24, 1.37]	0.70<br>[0.07, 1.2]	0.90<br>[0.66, 1.10]
s	0.04<br>[0.03,0.05]	0.10<br>[0.08, 0.11]	0.77<br>[0.43, 0.99]	0.04<br>[0.02, 0.05]	0.03<br>[0.02, 0.06]	0.03<br>[0.03, 0.05]
Tree height B.3	0.15<br>[0.14, 0.17]	0.19<br>[0.18, 0.20]	0.15<br>[0.14, 0.20]	0.18<br>[0.14, 0.25]	0.38<br>[0.15, 1.01]	0.34<br>[0.12, 0.80]
Substitution rate	*8E-4*	*8E-4*	*8E-4*	*8E-4*	1.6E-4<br>[3.5E-5, 3.1E-4]	1.3E-4<br>[2.7E-5, 3.8E-4]
UCLD standard deviation	-	-	-	1.40<br>[0.50, 2.20]	-	-

3. Simulations: Phylodynamic setup and summary statistics

In this case study, we used simulations to practically test if the method can recover dynamics of a superspreading event similar to the epidemiological context of Case study 1. We focus on two questions, representing first a negative and second a positive control: 1) Does our model setup combined with little-informative data produce elevated estimates of the reproductive number in absence of any transmission rate heterogeneity? 2) Can we recover the signal for an elevated reproductive number if it is elevated for only a very short time period directly after the start of the generative process? Below we shortly summarise the methods and results for both settings.

Supplementary text S1. Case study 1 - Superspreading: Simulations - phylodynamic setup

For both settings we used the BEAST2.6 package MASTER [1] to simulate trees from birth-death processes with sampling. To estimate the transmission dynamics, we used the package BDSKY [2].

Setting 1: Negative Control
We simulated 20 trees of 50 tips. Each tree was generated by a birth-death process with sampling defined by a reproductive number of 5, a rate to become non-infectious of 36.5y^{-1}

and a sampling proportion of 50%. All sequences were simulated along the respective tree using the HKY substitution model [3] with a kappa of 1.0, a substitution rate of 0.0008 substitutions/site/year [4] and equal base frequencies. For the re-inference, we allowed the reproductive number to change after 0.0082y, corresponding to 3 days, and set the sampling proportion to 0 until the date of the oldest sequence. We used the same prior distributions as in Sensitivity analysis 2, excluding the gamma rate categories. This setting aimed to explore if the wrongly assumed sampling proportion of 0 before the first sample combined with the very short first interval of the reproductive number can result in elevated estimates of the reproductive number in absence of any signal for it. As the results showed that we do not infer an elevated reproductive number in the first interval (Supplementary table S3), this setting suggests that the results presented in the manuscript are not generally an artefact from the model setup.

Setting 2: Positive Control
As above, we simulated 20 trees with 50 tips. In contrast, however, we defined a birth-death-sampling process with a reproductive number changing from 15 to 0.7 after 5 days. The rate to become non-infectious was set constant at 36.5 y^{-1} and the sampling proportion changes from 0 to 20% after 10 days. We generated nucleotide sequences with the HKY substitution model with a kappa of 1.0, a substitution rate of 0.0008 substitutions/site/year and equal base frequencies along each tree. We then used BDSKY to re-infer the parameters, fixing the change point of the reproductive number to the true value and assuming a constant sampling proportion with a Beta(200.0, 800.0) prior distribution. The remaining prior distributions and settings were set as in the main analysis, excluding the gamma rate categories. This setting was chosen to represent, as closely as possible, the epidemiological context of case study 1 and the parameters that we inferred in the main analysis. The good performance of the model supports that we should practically be able to estimate an increased reproductive number for a short time interval after the start of the process (Supplementary table S3).

Supplementary table S3. Case study 1 - Superspreading: Simulations - summary statistics. All 20 simulation replicates yielded ESS values above 200 for all the estimated parameters.

Parameter	Truth	Median	Relative error	Relative bias	Relative HPD width	95% HPD accuracy
Setting 1: Negative Control						
R_1	5.0	0.24	0.95	-0.95	1.19	86
R_2	5.0	4.63	0.1	-0.07	0.6	100
s_2	0.5	0.5	0.06	1E-4	1.86	100
Setting 2: Positive Control						
R_1	15.0	12.56	0.2	-0.16	0.46	71
R_2	0.7	3.75	5.59	4.36	6.22	93
s	0.2	0.2	0.003	-0.002	0.24	100

Case study 2 - Hospital outbreak

1. Main analysis: Phylodynamic setup and additional results

Supplementary text S2. Case study 2 - Hospital outbreak: Phylodynamic setup

In case study 2, we placed prior distributions for the sampling proportion parameters based on estimates given in [5], where surveillance sample genomes (N=320), collected between 16 August 2020 and 16 December 2020, represented 3.1% of all newly diagnosed PCR-test confirmed SARS-CoV-2 cases. In general, the percentage of sequenced surveillance cases of newly diagnosed infections per week ranged from 0 % up to 20 % (Supplementary table 1 in [5], supplementary figure S2 below). The proportion of sampled cases fluctuated principally over the first nine weeks of the sampling period, i.e. calendar weeks 33–41. During this period, the percentage of all sequenced cases over all confirmed infections was 11.6% (171 sequenced samples / 1469 cases). Throughout the next 10 weeks, i.e. calendar weeks 42–51, the overall percentage of sequenced cases stayed below three percent. As a result, we decided to allow the sampling proportion to change in a piecewise fashion on 12 October 2020, the start of calendar week 42 and set Uniform(0.0, 0.20) and Beta(3.1, 96.9) prior distributions for $s_{surveillance,t1}$ and $s_{surveillance,t2}$, respectively. The SARS-CoV-2 outbreak at Düsseldorf University Hospital Ward D led to identification of 29 positive cases and through subsequent targeted sequencing efforts viral genomes were reconstructed from 23 infected individuals [5]. We utilised the ratio of sequenced vs. all identified cases (23/29) to set the upper bound for the hospital sampling proportion prior. Since unreported asymptomatic infections may constitute a significant portion of all transmissions, we chose to account for the potential undiagnosed cases by selecting a Uniform(0.0, 0.8) prior distribution for $s_{t1,hospital}$.

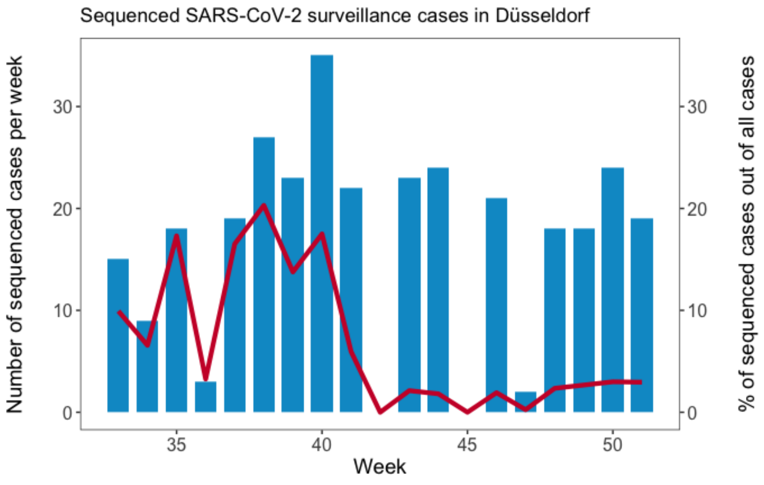

Supplementary figure S2. Case study 2 - Hospital outbreak: Number of sequenced surveillance samples in Düsseldorf between weeks 33 and 51 in 2020. Blue histograms represent the absolute number of sequenced cases per week (y axis on the left) whereas the red line represents the percentage of sequenced cases per week (y axis on the right). In total, sequenced surveillance samples (N=320), covered 3.1% of all newly diagnosed SARS-CoV-2 cases. Substantial drop in percentage of sequenced cases occurred during the calendar

week 42, and therefore within Bayesian inference we allowed a piecewise change in the surveillance sampling proportion on 12 October 2020. Figure created according to Supplementary table S1 in [5].

Supplementary table S4. Case study 2 - Hospital outbreak: Overview of prior distributions used. As described in the main text, we fixed the rate to become non-infectious to δ=36.5 years^{-1}. Additionally, for both clock models the substitution rate was fixed to 0.0008 substitutions/site/year. Moreover, $r_{\lambda,surveillance}$ was fixed to its true value of 1.0. For κ, gamma shape s, the base frequencies, and UCLD standard deviation we applied the default prior distributions.

Parameter	Main analysis	Sensitivity analysis 1 (Wide uniform prior on $s_{surveillance,t1}$)	Sensitivity analysis 2 (Uniform prior on $s_{surveillance,t2}$)	Sensitivity analysis 3 (UCLD relaxed clock)
$R_{base,t1}$	LN(0.0, 1.25)	LN(0.0, 1.25)	LN(0.0, 1.25)	LN(0.0, 1.25)
$R_{base,t2}$	LN(0.0, 1.25)	LN(0.0, 1.25)	LN(0.0, 1.25)	LN(0.0, 1.25)
$r_{\lambda,hospital}$	LN(0.0, 1.0)	LN(0.0, 1.0)	LN(0.0, 1.0)	LN(0.0, 1.0)
$s_{surveillance,t1}$	Unif(0.0, 0.2)	Unif(0.0, 1.0)	Unif(0.0, 0.20)	Unif(0.0, 0.20)
$s_{surveillance,t2}$	Beta(3.1, 96.9)	Beta(3.1, 96.9)	Unif(0.0, 0.2)	Beta(3.1, 96.9)
$s_{t1,hospital}$	Unif(0.0, 0.8)	Unif(0.0, 0.8)	Unif(0.0, 0.8)	Unif(0.0, 0.8)
κ	LN(1.0,1.25)	LN(1.0,1.25)	LN(1.0,1.25)	LN(1.0,1.25)
gamma shape s	Exp(1.0)	Exp(1.0)	Exp(1.0)	Exp(1.0)
Base frequencies	Unif(0.0,1.0)	Unif(0.0,1.0)	Unif(0.0,1.0)	Unif(0.0,1.0)
UCLD standard deviation	–	–	–	Gamma(0.5396, 0.3819)

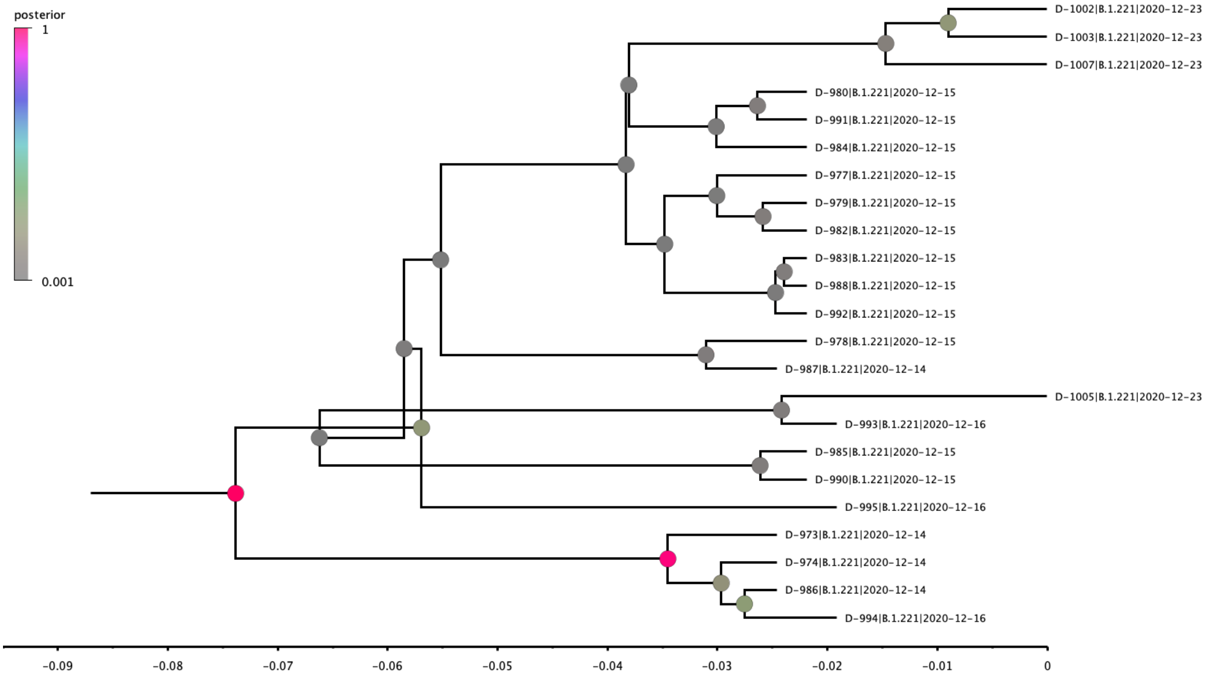

Supplementary figure S3. Case study 2 - Hospital outbreak: MCC tree for the hospital cluster. Maximum clade credibility (MCC) tree reconstructed based on viral genomes obtained from a nosocomial outbreak (‘Ward D’, N=23). Colouring of the nodes’ circles represents the posterior values: grey colours indicate nodes with low posterior support whereas branching events with high support are indicated with fuchsia. The clonal nature of the SARS-CoV-2 outbreak at Ward D means that the viral genomes share identical or nearly identical genetic profiles as shown in Figure 3C in [5]. This leads to low posterior support for some branching events within the phylodynamic tree further enabling negative branch lengths when choosing ‘median node heights’ for MCC tree reconstruction. Tree was visualised with FigTree v1.4.4 (http://tree.bio.ed.ac.uk/software/figtree/, last visited 2024-06-24).

2. Sensitivity analyses: Summary statistics

Supplementary text S3. Case study 2 - Hospital outbreak: Sensitivity analyses phylodynamic setup

To evaluate the sensitivity of the inferred R_{base} and $r_{\lambda,hospital}$ estimates for the surveillance sampling proportion prior distribution used, we performed two additional analyses (Supplementary table S5). In the first setting, we used Uniform(0.0, 1.0) as a prior distribution for $s_{surveillance,t1}$ whereas in the second sensitivity analysis, we used a Uniform(0.0, 0.2) prior distribution for $s_{surveillance,t2}$. For both analyses the other prior distributions were placed as described above. To further investigate the impact of assuming strictly clock-like evolution, we conducted an additional sensitivity analysis using the uncorrelated lognormal relaxed clock model [6]. We fixed the mean rate of the relaxed clock model to 0.0008 substitutions/site/year and estimated the standard deviation. The prior distribution for the standard deviation was set to the default Gamma distribution with shape parameter 0.5396 and rate parameter 0.3819.

Supplementary table S5. Case study 2 - Hospital outbreak: Sensitivity analyses - Overview of inferred posterior distributions for the main parameters of interest. Median estimates with 95% HPDIs are presented. In sensitivity analysis 1, we used a Uniform(0.0, 1.0) prior distribution for $s_{surveillance,t1}$. In sensitivity analysis 2, we used a Uniform(0.0, 0.2) prior distribution for $s_{surveillance,t2}$. In sensitivity analysis 3, we employed an uncorrelated lognormal relaxed clock model.

Parameter	Main analysis	Sensitivity analysis 1 (Wide uniform prior on $s_{surveillance,t1}$)	Sensitivity analysis 2 (Uniform prior on $s_{surveillance,t2}$)	Sensitivity analysis 3 (UCLD relaxed clock)
$R_{base,t1}$	1.15 [0.97, 1.35]	1.15 [0.97, 1.35]	1.14 [0.95, 1.34]	1.15 [0.95, 1.35]
$R_{base,t2}$	1.48 [1.21, 1.76]	1.48 [1.20, 1.77]	1.44 [1.15, 1.73]	1.50 [1.20, 1.77]
$r_{\lambda,hospital}$	1.44 [0.85, 2.26]	1.42 [0.85, 2.20]	1.47 [0.84, 2.30]	1.36 [0.79, 2.15]
$s_{surveillance,t1}$	0.10 [0.05, 0.18]	0.11 [0.04, 0.19]	0.11 [0.05, 0.19]	0.11 [0.06, 0.19]
$s_{surveillance,t2}$	0.04 [0,02, 0.06]	0.04 [0.01, 0.06]	0.04 [0.02, 0.08]	0.03 [0.01, 0.06]
$s_{hospital}$	0.72 [0.50, 0.80]	0.72 [0.52, 0.80]	0.72 [0.51, 0.80]	0.71 [0.50, 0.80]

3. Simulations: Phylodynamic setup and summary statistics

Supplementary text S4. Case study 2 - Hospital outbreak: Simulations - phylodynamic setup

To further evaluate the performance of the BDSKYλ model [7] under transmission dynamics similar to those of case study 2, we conducted an additional simulation study. We used MASTER to simulate two transmission trees: one representing the 'surveillance' data and the other representing the 'hospital outbreak'. We set the transmission parameters to match the empirical data, using values of R_{base}=1.5, $r_{\lambda,surveillance}$=1.0, $r_{\lambda,hospital}$=1.4, $s_{surveillance}$=0.07, $s_{hospital}$ =0.7, and δ=36.5y^{-1}. For simplicity, we omitted the piecewise decline for R_{base} in the simulations. Each simulation was run until a specific number of tips was reached: 50 for the surveillance tree, matching the empirical data, and 25, 10, or 5 for the hospital tree, to evaluate the model performance under smaller-scale hospital outbreaks. We also delayed the onset of the sampling process for the surveillance tree by 28 days, corresponding to the empirical data for which we observed approximately a four-week time interval between the median estimate of the tree height and the first sampling date. We simulated sequence data along the transmission trees using the HKY model, assuming a rate of 0.0008 substitutions/site/year and a sequence length of 30,000bp. For each scenario with a different number of requested tips from the hospital outbreak (25, 10, or 5), we performed 20 simulation replicates. For the

Bayesian inference, we fixed the become non-infectious rate and clock rate to their true values, and estimated the transmission rate ratio $r_{\lambda,hospital}$, while fixing $r_{\lambda,surveillance}$ to 1.0. We used the same prior distributions as in the main analysis of the empirical data.

The results of the simulation study are presented in Supplementary table S6, where we evaluate the performance of the BDSKYλ model using the following metrics for each parameter of interest: median, relative error, relative bias, relative highest posterior density interval (HPDI) width, and 95% HPD accuracy. Under each scenario, the median estimates for R_{base} and $r_{\lambda,hospital}$ are close to the true simulated values, with 95% HPD accuracy estimates ranging from 90% to 100%. However, the relative HPDI width for $r_{\lambda,hospital}$ increases as the number of samples from the hospital outbreak decreases, indicating lower precision of the model. In each scenario, the median estimates for $s_{surveillance}$ and $s_{hospital}$ are somewhat underestimated compared to the true simulated values, despite high 95% HPD accuracy (75–100%). Additionally, the relative HPD width for $s_{hospital}$ increases as the number of samples from the hospital outbreak decreases. Nevertheless, overall, these results suggest that under simulation settings reflecting the empirical data, the BDSKYλ model is capable of inferring the parameters of interest robustly, even when the number of samples from the hospital outbreak is limited to five.

Supplementary table S6. Case study 2 - Hospital outbreak: Results from the simulation study. For the Bayesian inference, the transmission rate ratio $r_{\lambda,surveillance}$ was fixed to its true value ($r_{\lambda,surveillance} = 1.0$). For all scenarios with varying number of tips requested from the hospital ($N_{tips} = 25, 10, 5$), all 20 simulation replicates yielded ESS > 200 for all the parameters included in the model.

Hospital N_{tips}	Parameter	Truth	Median	Relative error	Relative bias	Relative HPD width	95% HPD accuracy
$N_{tips} = 25$	R_{base}	1.5	1.54	0.04	0.02	0.26	95
	$r_{\lambda,surveillance}$	1.0	1.0 (fixed)	-	-	-	-
	$r_{\lambda,hospital}$	1.4	1.39	0.12	-0.01	0.81	95
	$s_{surveillance}$	0.07	0.05	0.29	-0.29	0.80	75
	$s_{hospital}$	0.7	0.62	0.18	-0.11	1.01	100
$N_{tips} = 10$	R_{base}	1.5	1.53	0.06	0.02	0.27	95
	$r_{\lambda,surveillance}$	1.0	1.0 (fixed)	-	-	-	-
	$r_{\lambda,hospital}$	1.4	1.63	0.28	0.17	1.43	95
	$s_{surveillance}$	0.07	0.06	0.21	-0.18	0.86	100
	$s_{hospital}$	0.7	0.56	0.28	-0.20	1.14	100
$N_{tips} = 5$	R_{base}	1.5	1.55	0.07	0.04	0.27	90
	$r_{\lambda,surveillance}$	1.0	1.0 (fixed)	-	-	-	-
	$r_{\lambda,hospital}$	1.4	1.47	0.24	0.05	1.86	100
	$s_{surveillance}$	0.07	0.05	0.28	-0.23	0.84	90
	$s_{hospital}$	0.7	0.54	0.23	-0.23	1.26	100

Case study 3 - Spatiotemporal pandemic excerpt

1. Data: Generation and metadata

Supplementary text S5. Case study 3 - Spatiotemporal pandemic excerpt: Data generation

The Department of Infection Prevention and Infectious Diseases of the University Medical Center Regensburg and the MVZ Laboratory Passau of the Limbach Group were participating in the sequencing of SARS-CoV-2 viruses according to the Regulation on Molecular Surveillance of Coronavirus SARS-CoV-2 of the Federal Ministry of Health in Germany (https://robert-koch-institut.github.io/SARS-CoV-2-Sequenzdaten_aus_Deutschland/ last visited 2024-06-17). Genomes that were collected between January 13, 2021 and October 20, 2021, originated from the eastern Bavarian region and sequenced as part of this project were provided as a data set for the present study.

At the University Medical Center Regensburg, the Freed and Silander protocol (https://dx.doi.org/10.17504/protocols.io.bwyppfvn, last visited 2024-06-18) was followed for sequencing. In brief, upon RNA extraction and cDNA synthesis, 1200bp amplicons were generated using the SARS-Cov2-midnight-1200 primer set (10007184, IDT), followed by barcoding using the rapid barcoding kit 96 (SQK-RBK110.96, Oxford Nanopore). Sequencing and base calling was performed for ~15h using the MK1c device. For the analysis of the nanopore data we use the poreCov pipeline (depending on the date of sequencing in the latest available version [8]).

An Illumina NextSeq 550 (Illumina Inc., San Diego, CA, USA) was used for sequencing in the MVZ Passau laboratory in combination with the CovidSeq Kit from Illumina (Illumina, San Diego, CA, USA) or the EasySeq™ SARS-CoV-2 WGS NGS Library Prep Kit from NimaGen (NimaGen, Nijmegen, Netherlands), depending on availability. The CovidSeq Kit included all necessary reagents for SARS-CoV-2 whole-genome sequencing, while the EasySeq™ SARS-CoV-2 WGS NGS Library Prep Kit provided a streamlined workflow for whole-genome sequencing of SARS-CoV-2. The CLC Genomics Workbench 20.0 (Qiagen, United States) was used to assemble the genomes.

In supplementary figure S4 we provide a visual representation of the spatial distribution of the sequence samples next to distribution of COVID-19 case counts in the same time period.

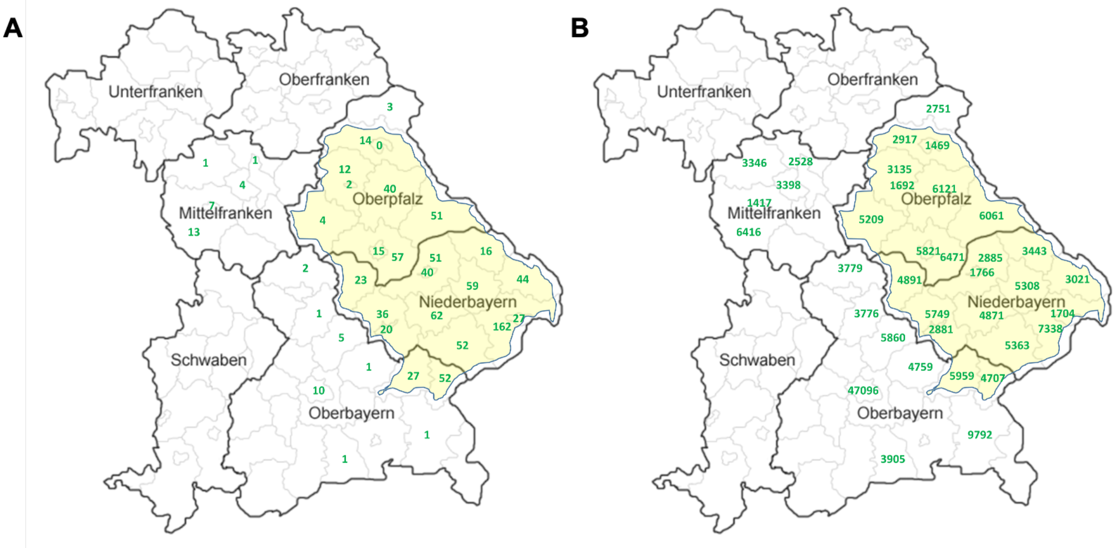

Supplementary figure S4. Case study 3 - Spatiotemporal pandemic excerpt: Sequence and diagnosis counts. Spatially resolved number of genome sequences included in the data set (panel A) and diagnosed infections (panel B) between 13 January 2021 and 30 October 2021. Data source: https://www.lgl.bayern.de/gesundheit/infektionsschutz/infektionskrankheiten_a_z/coronavirus/karte_coronavirus/archiv.htm

2. Main analysis: Prior settings and additional results

Below we provide a table summarising all prior probability distributions on model parameters.

Supplementary table S7. Case study 3 - Spatiotemporal pandemic excerpt: Prior distributions for all analyses. We fixed the rate to become non-infectious to 36.5 y^{-1}, the clock rate to 0.0008 substitutions/site/year, the sampling proportion of type 2 to 0.0, the geo frequencies to 0.0 for type 1 and 1.0 for type 2 and restricted the migration rates to be symmetric. For κ, gamma shape s, and the base frequencies we applied the default prior distributions.

Parameter	Main analysis	Sensitivity analysis 1 (seven R intervals)	Sensitivity analysis 2 (min.20 sequences)	Sensitivity analysis 3 (VOC stratification)
$R^1_{e,i}$	LN(0.0, 4.0)	LN(0.0, 4.0)	LN(0.0, 4.0)	LN(0.0, 4.0)
$R^2_{e,i}$	LN(0.0, 4.0)	LN(0.0, 4.0)	LN(0.0, 4.0)	LN(0.0, 4.0)
s^1_i	Beta(50.0, 950.0)	Beta(50.0, 950.0)	Beta(50.0, 950.0)	Beta(50.0, 950.0)
Migration rate m	Exp(1.0)	Exp(1.0)	Exp(1.0)	Exp(1.0)
κ	LN(1.0,1.25)	LN(1.0,1.25)	LN(1.0,1.25)	LN(1.0,1.25)
Gamma shape s	Exp(1.0)	Exp(1.0)	Exp(1.0)	Exp(1.0)
Base frequencies	Unif(0.0,1.0)	Unif(0.0,1.0)	Unif(0.0,1.0)	Unif(0.0,1.0)

3. Sensitivity analyses: Summary statistics

Below we provide visualisations for the summary statistics of all three sensitivity analyses.

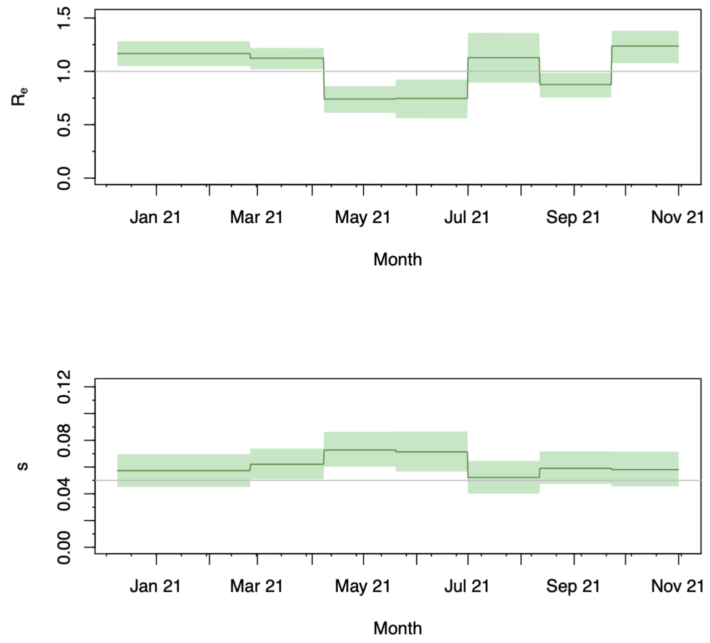

Supplementary figure S5. Case study 3 - Spatiotemporal pandemic excerpt: Sensitivity analysis with six change points. Posterior estimates of the effective reproductive number (R_e) and sampling proportion (s) with six change points through time (2021-02-24, 2021-04-07, 2021-05-19, 2021-06-30, 2021-08-11, 2021-09-22). The bold line corresponds to the inferred median, the shaded area to the 95% HPDI and the grey horizontal line to an R_e of 1 (upper panel) and to the prior mean of 5% (lower panel), respectively.

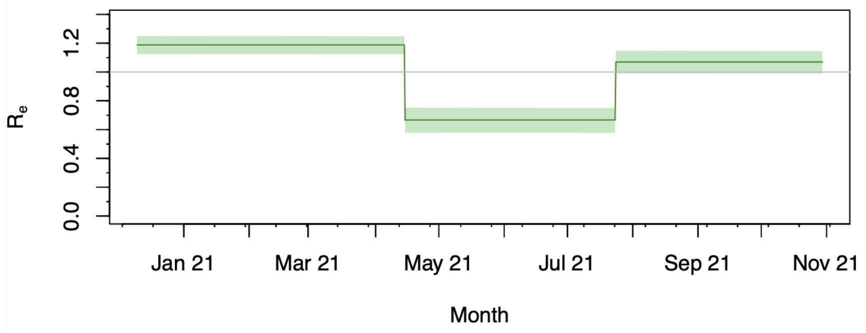

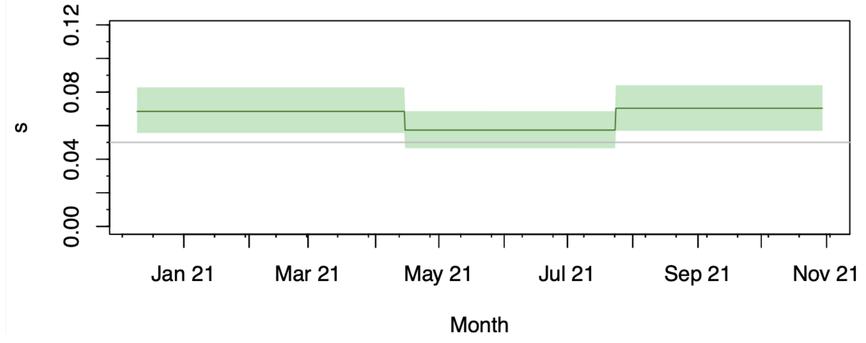

Supplementary figure S6. Case study 3 - Spatiotemporal pandemic excerpt: Sensitivity analysis with different cluster exclusion threshold. Posterior estimates of the effective reproductive number (R_e) and sampling proportion (s) based on Pango lineages with at least 20 sequences. The bold line corresponds to the inferred median, the shaded area to the 95% HPDI and the grey horizontal line to an R_e of 1 (upper panel) and to the prior mean of 5% (lower panel), respectively.

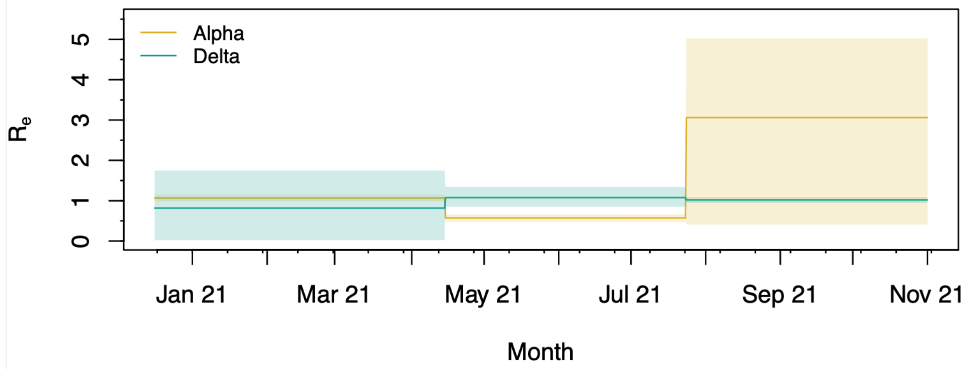

Supplementary figure S7. Case study 3 - Spatiotemporal pandemic excerpt: Sensitivity analysis with stratification by VOC. Posterior estimates of the effective reproductive number (R_e) for Pango lineages with at least 5 sequences belonging to the Alpha variant (yellow) and Delta variant (green). The bold line corresponds to the inferred median, the shaded area to the 95% HPDI.

4. Simulations: Phylodynamic setup and summary statistics

As the multi-type birth-death model allows for many different model variations and can thus be adapted to the setup of interest in each study, we opted to first test the performance on synthetic data simulated under our model. Specifically, we aimed to test the accuracy of estimates of the effective reproductive number of the sampled type.

Supplementary text S6. Case study 3 - Spatiotemporal pandemic excerpt: Simulations setup

We used the package MASTER implemented in BEAST2 to simulate 20 trees with 100 tips under a constant-rate birth-death-sampling model with two types. We set a birth rate of $73.0y^{-1}$, death rate of $33.675y^{-1}$ and sampling rate of $1.825y^{-1}$ for type one. Type two was assigned a birth rate of $54.75y^{-1}$, death rate of $36.5y^{-1}$ and sampling rate of $0.0y^{-1}$. This corresponds to an effective reproductive number (R_e) of 2.0 (type one) and 1.5 (type two), a sampling proportion (s) of 5% (type one) and 0% (type two) as well as a rate to become non-infectious of $36.5y^{-1}$. Type changes ('migration') are allowed with a rate of $0.12y^{-1}$ in both directions. Along each tree we simulated sequences of 30,000 base pairs under a HKY substitution model and strict clock model with a rate of 0.0008 substitutions/site/year. For each of the resulting 20 sequence alignments we then set up a separate BDMM analysis under the simulated model, however, estimating the effective reproductive number for both types, the sampling proportion, type-change rate, tree topology and branch lengths. The migration rates are assumed to be symmetric. We fix the rate to become non-infectious and the substitution rate to the correct value and the geo-frequencies to 1 for the unsampled and 0 for the sampled compartment. We set a Lognormal(0.0,1.25) distribution as prior on the effective reproductive number and an Exponential distribution with mean 1.0 for the migration rate. For the other parameters we used the default priors, including a uniform prior between 0 and 1 for the sampling proportion. We ran all MCMC chains for $2 * 10^7$ steps. The results are visualised in Supplementary figure S8. The good HDPI accuracy and mostly also narrow HPD width indicate that the model parameters can, in the considered setup, be practically estimated.

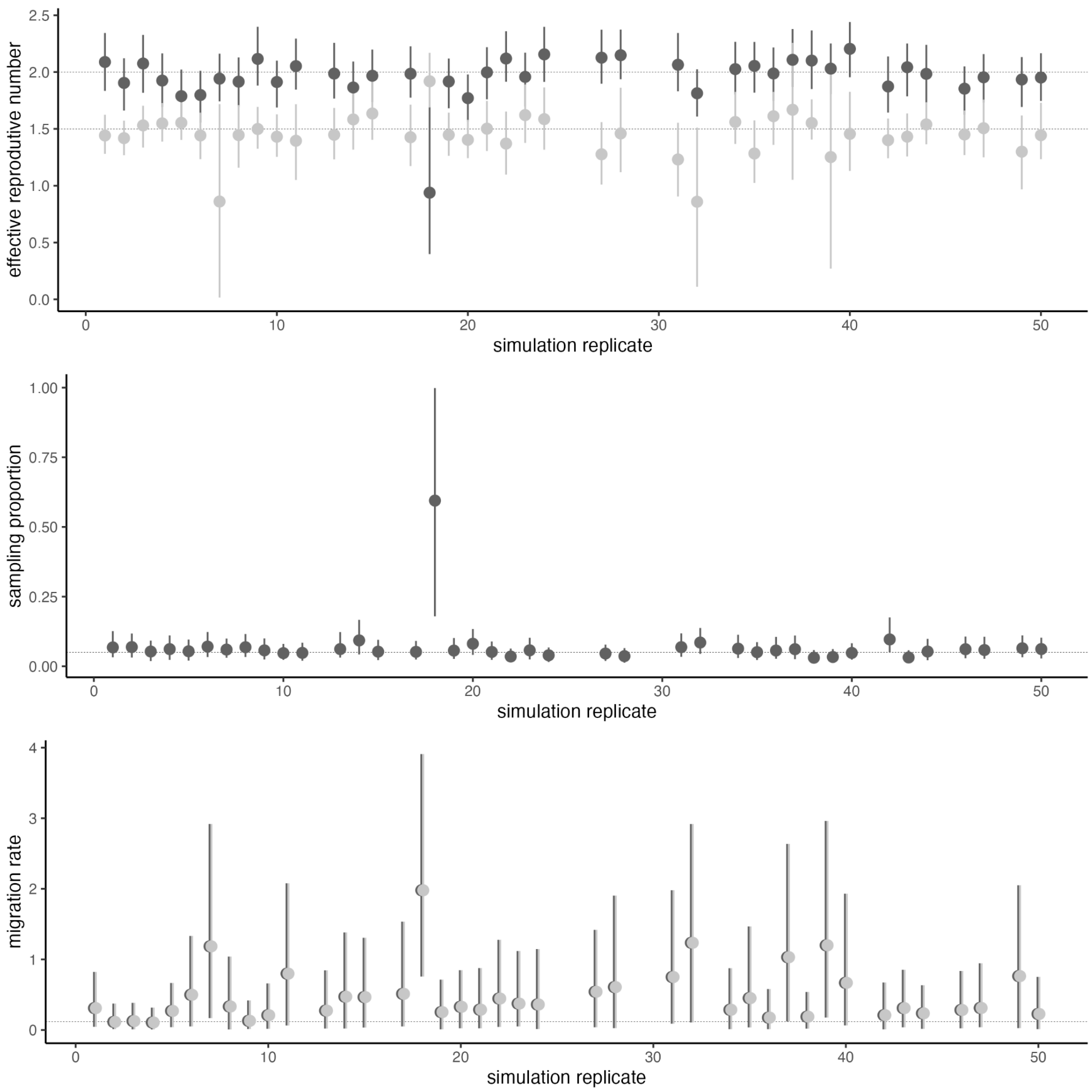

Supplementary figure S8. Case study 3 - Spatiotemporal pandemic excerpt: Effective reproductive number (upper panel), sampling proportion (middle panel) and migration rate (lower panel) estimated from simulated sequence data as described above. The dotted line corresponds to the value used for simulation. Each vertical line shows the 95% HPDI of a simulation replicate for which all estimated parameters showed ESS values of at least 200 (40/50); median estimate is highlighted as dot. Estimates for the sampled population are shown in dark grey, for the unsampled population in light grey.

References

1. Vaughan TG, Drummond AJ. A stochastic simulator of birth-death master equations with application to phylodynamics. *Molecular Biology and Evolution*. 2013;30(6):1480-1493. doi:10.1093/molbev/mst057

2. Stadler T, Kühnert D, Bonhoeffer S, Drummond AJ. Birth-death skyline plot reveals temporal changes of epidemic spread in HIV and hepatitis C virus (HCV). *Proceedings of the National Academy of Sciences of the United States of America*. 2013;110(1):228-233. doi:10.1073/pnas.1207965110

3. Hasegawa M, Kishino H, Yano T aki. Dating of the human-ape splitting by a molecular clock of mitochondrial DNA. *Journal of Molecular Evolution*. 1985;22(2):160-174. doi:10.1007/BF02101694

4. Ghafari M, Du Plessis L, Pybus OG, Katzourakis A. Time dependence of SARS-CoV-2 substitution rates. Virological. October 27, 2020. Accessed December 5, 2022. https://virological.org/t/time-dependence-of-sars-cov-2-substitution-rates/542

5. Walker A, Houwaart T, Finzer P, et al. Characterization of Severe Acute Respiratory Syndrome Coronavirus 2 (SARS-CoV-2) Infection Clusters Based on Integrated Genomic Surveillance, Outbreak Analysis and Contact Tracing in an Urban Setting. *Clinical Infectious Diseases*. 2022;74(6):1039-1046. doi:10.1093/cid/ciab588

6. Drummond AJ, Ho SYW, Phillips MJ, Rambaut A. Relaxed phylogenetics and dating with confidence. *PLoS Biology*. 2006;4(5):699-710. doi:10.1371/journal.pbio.0040088

7. Weber A, Översti S, Kühnert D. Reconstructing relative transmission rates in Bayesian phylodynamics: Two-fold transmission advantage of Omicron in Berlin, Germany during December 2021. *Virus Evolution*. 2023;9(2):vead070. doi:10.1093/ve/vead070

8. Brandt C, Krautwurst S, Spott R, et al. poreCov-An Easy to Use, Fast, and Robust Workflow for SARS-CoV-2 Genome Reconstruction via Nanopore Sequencing. *Front Genet*. 2021;12. doi:10.3389/fgene.2021.711437
